# Selection of candidate genes affecting meat quality and preliminary exploration of related molecular mechanisms in the Mashen pig

**DOI:** 10.5713/ajas.18.0718

**Published:** 2019-02-14

**Authors:** Pengfei Gao, Zhimin Cheng, Meng Li, Ningfang Zhang, Baoyu Le, Wanfeng Zhang, Pengkang Song, Xiaohong Guo, Bugao Li, Guoqing Cao

**Affiliations:** 1 College of Animal Science and Veterinary Medicine, Shanxi Agricultural University, Taigu 030801, China

**Keywords:** Pig, Candidate Gene, Molecular Mechanism, Meat Quality, RNA-Seq

## Abstract

**Objective:**

The aim of this study was to select the candidate genes affecting meat quality and preliminarily explore the related molecular mechanisms in the Mashen pig.

**Methods:**

The present study explored genetic factors affecting meat quality in the Mashen pig using RNA sequencing (RNA-Seq). We sequenced the transcriptomes of 180-day-old Mashen and Large White pigs using longissimus dorsi to select differentially expressed genes (DEGs).

**Results:**

The results indicated that a total of 425 genes were differentially expressed between Mashen and Large White pigs. A gene ontology enrichment analysis revealed that DEGs were mainly enriched for biological processes associated with metabolism and muscle development, while a Kyoto encyclopedia of genes and genomes analysis showed that DEGs mainly participated in signaling pathways associated with amino acid metabolism, fatty acid metabolism, and skeletal muscle differentiation. A MCODE analysis of the protein-protein interaction network indicated that the four identified subsets of genes were mainly associated with translational initiation, skeletal muscle differentiation, amino acid metabolism, and oxidative phosphorylation pathways.

**Conclusion:**

Based on the analysis results, we selected glutamic-oxaloacetic transaminase 1, malate dehydrogenase 1, pyruvate dehydrogenase 1, pyruvate dehydrogenase kinase 4, and activator protein-1 as candidate genes affecting meat quality in pigs. A discussion of the related molecular mechanisms is provided to offer a theoretical basis for future studies on the improvement of meat quality in pigs.

## INTRODUCTION

Pigs are widely used in meat production, constitute a main source of animal protein, and are a key economic resource. Over the past 2 decades, intensive selection of western pig breeds has led to the generation of animals with rapid and effective muscle growth. Yet, these features have been associated with the deterioration of meat quality [1,2]. With the improvement of living standards and increased consumer consciousness, a high lean meat percentage is no longer the only requirement for pork; instead, modern consumers tend to select pork that is both high quality and healthy. For this reason, the simultaneous guarantee of growth rate and meat quality has become an important goal in the pig industry. Skeletal muscle is the main flesh-producing tissue in pigs and accounts for approximately 40% of body weight. Muscle fiber number and diameter are important factors that affect muscle traits. In addition to muscle fiber type, intramuscular fat content within skeletal muscle also has a major effect on meat quality indicators such as flavor, external appearance, and tenderness [3].

RNA sequencing (RNA-Seq) is a transcriptomic method based on next-generation sequencing technologies that enables the accurate measurement of RNA expression levels in tissue. Comprehensive transcriptome analyses can accelerate the systematic understanding of gene expression, regulation, and networks. Given a variety of advantages, RNA-Seq technology has been widely applied to study the muscle transcriptomes of livestock [4,5]. For example, Huang et al [6] used RNA-Seq technology to select differentially expressed genes (DEGs) in the subcutaneous and intramuscular adipose tissues of Large White pigs and found that these genes were mainly involved in lipid metabolism, regulated adipocyte differentiation through the MAPK signaling pathway, and accordingly affected lipid accumulation in subcutaneous and intramuscular adipose tissues. In another study, Xu et al [7] sequenced the transcriptome of skeletal muscle tissue from Mashen pigs at 65 days of fetal age and at 3, 60, and 120 days of postnatal age and identified 338 DEGs. The results of a functional analysis revealed that DEGs were mainly associated with metabolism, myofibril formation, the cytoskeleton, contractile activity, and signal transduction.

The Large White pig is a classic western lean hog breed that is renowned for its high lean meat percentage and rapid growth rate and is widely used in the production of commercial pigs [8]. In contrast, the Mashen is a Chinese domestic breed that has a lower growth rate, lower feed conversion efficiency, and a lower lean meat percentage compared to western breeds. Yet, the Mashen offers several advantages over western breeds in terms of genetic diversity, reproductive capability, intramuscular fat content, and meat quality. In a study by Zhao et al [9], Large White pigs had a significantly higher growth rate than Mashen pigs. Zhao et al [10] found that the number and volume of adipocytes in the longissimus dorsi muscle were significantly higher in 180-day-old Mashen pigs compared to age-matched Large White pigs. Similarly, mRNA and protein levels of adipogenesis markers such as CCAAT enhancer binding protein beta (C/EBPβ), peroxisome proliferator activated receptor gamma (PPARγ), and fatty acid binding protein 4 (FABP4) were significantly higher in Mashen pigs than in Large White pigs. Therefore, the Mashen and Large White pigs are ideal breeds for studying growth performance and meat quality [11]. A sequencing analysis of the longissimus dorsi muscles of different pig breeds at the transcriptome level not only informs breed-specific gene transcription and regulation patterns as they relate to muscle growth and development, but can also assist the characterization of the genetic mechanisms of growth and meat quality differences in pigs [12,13]. Therefore, we selected 180-day-old Large White and Mashen pigs for transcriptome sequencing of longissimus dorsi muscle tissue using RNA-Seq technology for the selection of DEGs and the identification of regulatory networks relevant to muscle growth and development. The results of this study can serve as a reference for an analysis of genetic mechanisms that influence growth, development, and meat quality in pigs, and can also provide a theoretical basis for the enhancement of growth performance and meat quality.

## MATERIALS AND METHODS

### Experimental materials

Three Large White pigs and three Mashen pigs aged 180 days were obtained from the Datong Pig Farm in Shanxi Province. All pigs were reared in the same environment under identical nutritional conditions. The experiment was performed in accordance with the Charter of the Animal Ethics Committee of Shanxi Agricultural University and was approved by the Shanxi Agricultural University. After slaughter, longissimus dorsi muscle samples were collected, labeled as L61, L62, L63, M61, M62, and M63 (L, Large White; M, Mashen), snap frozen in liquid nitrogen, transported back to the laboratory, and stored at −80°C until use.

### RNA extraction, cDNA library construction, and Illumina sequencing

Total RNA was extracted using TRIzol Reagent (Thermo Fisher Scientific, Carlsbad, CA, USA). The obtained RNA was further purified using the RNeasy Kit (QIAGEN GmbH, Hilden, Germany) and RNA integrity was assessed using an Agilent 2100 Bioanalyzer (Agilent, Santa Clara, CA, USA). Subsequently, cDNA libraries were constructed using the TruSeq Rapid Duo cBot Sample Loading Kit as per manufacturer instructions with the construction process including the isolation of mRNA with Oligo(dT) magnetic beads, RNA fragmentation, cDNA synthesis, and polymerase chain reaction (PCR) amplification. Raw reads were then generated by 2×100 bp paired-end sequencing of the prepared cDNA on an Illumina HiSeq 2500 system (Illumina, Inc., San Diego, CA, USA).

### Quality control

Clean reads were obtained by quality control of the raw reads using Perl scripts that removed adapter sequences, sequences with unknown nucleic sequence information >10%, and redundant sequences with >50% of bases having quality scores of ≤10.

### Selection of differentially expressed genes

A directory of clean reads was created by running Bowtie2 (v2.2.9) on the server and the sequence reads obtained after quality control were mapped to the pig genome using TopHat (v2.0.12; http://hgdownload.soe.ucsc.edu/goldenPath/susScr3/bigZips/susScr3.fa.gz). Known transcripts were named with Ensembl IDs while unknown transcripts were named with TCON IDs. The reads were then assembled into a new GTF file using Cufflinks (v2.2.1) and Cuffmerge (v3), and subsequent screening of the transcript information was performed using Cuffdiff (v2.0.2). The fragments per kilobase of exon per million mapped reads method was used to normalize transcript expression levels. Finally, transcripts with differential expression were selected using the following screening criteria: p<0.01, false discovery rate (FDR) <0.05, and |log2(fold change [FC])|≤1.

### Functional annotation of transcripts

Gene ontology (GO) and Kyoto encyclopedia of genes and genomes (KEGG) annotations were applied to known transcripts. For unknown transcripts, screening was first performed using the criteria of transcript length ≥200 bp and number of exons ≥2; then, BLAST was used to compare the pre-translated sequences of the unknown transcripts with the protein sequence databases non-redundant nucleotide sequence database, non-redundant protein sequence database, and SwissProt based on the same screening criteria for DEGs (p<0.01). Subsequently, DAVID was used to perform GO and KEGG annotations and to consolidate annotation information for known and unknown transcripts.

### Protein-protein interaction network analysis

The interaction network for known genes was obtained from the STRING database and imported into Cytoscape for visualization. Nodes and modules of the gene network imported into Cytoscape (k-core = 4) were then analyzed for the identification of core genes using MCODE [14].

### Validation of differentially expressed genes by quantitative real-time polymerase chain reaction

Quantitative real-time PCR (qRT-PCR) was used to validate the transcription levels of DEGs. A quantity of 0.5 μg of RNA extracted from each muscle tissue sample was reverse transcribed into a cDNA template. The qRT-PCR was then performed using the SYBR PrimixScript RT-PCR Kit (TaKaRa, Tokyo, Japan). The reaction system consisted of the following: 1 μL cDNA, 0.2 μL forward primer, 0.2 μL reverse primer, 5 μL SYBR Premix Ex Taq (2×), and 3.6 μL RNase free H_2_O. The PCR procedure was as follows: pre-denaturation at 95°C for 30 s, denaturation at 95°C for 10 s, and renaturation and extension at 60°C for 30 s, 40 cycles. The specific primers used for qRT-PCR are shown in Table 1, including primers for 18S rRNA as a reference gene. Relative mRNA expression levels were calculated using the 2^−ΔΔCt^ method and the t-test in SPSS was used for statistical analysis of the relative expression levels (p<0.01).

## RESULTS

### Sequencing results

A total of 98.7 Gb of raw data were obtained from six cDNA libraries after deep sequencing with the Illumina HiSeq 2500 system, and 93.4 Gb of clean data were obtained after quality control. A total of 57 Gb of raw data were obtained from the muscle tissues of Large White pigs (L6, including L61, L62, and L63). After filtering adapter sequences and low-quality reads, a total of 50.4 Gb of clean data were obtained with Q20> 95.90% and Q30>86.40%. A total of 45.3 Gb of raw data were obtained from the muscle tissues of Mashen pigs (M6, including M61, M62, and M63). After filtering, 43 Gb of clean data were obtained with Q20>96% and Q30>87.10% (Table 2). Approximately 68% of reads were mapped solely to the reference pig genome; the overall mapping rate of the samples was 69.52% (Table 3). Based on these results, it was deduced that the generated sequencing data were of high quality and usable for subsequent analyses.

### Differentially expressed genes

**Figure 1:**
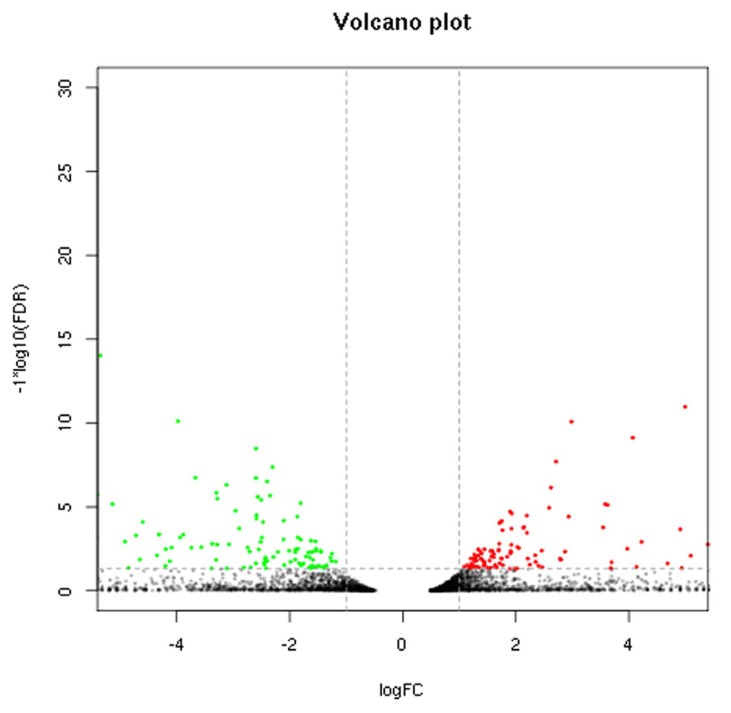
Volcano plot of differentially expressed genes in Mashen and Large White pigs. The points below of the “—” denotes genes with no significant changes; The points on the right of the “|” denotes up-regulated genes; The points on the left of the “|” denotes down-regulated genes.

**Figure 2:**
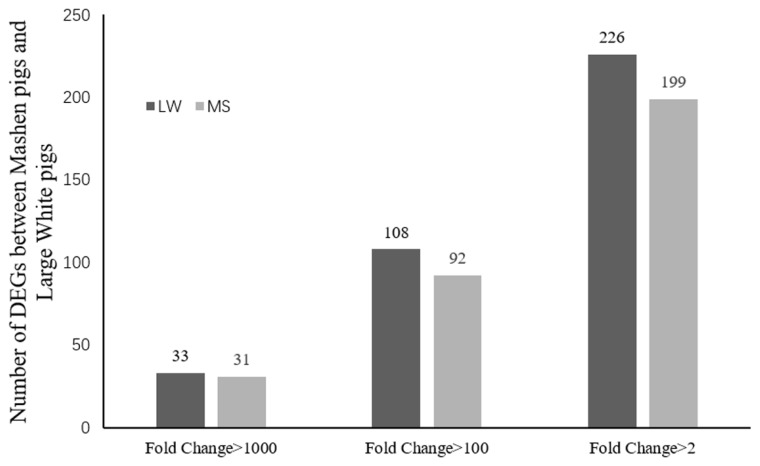
The number of up-regulated and down-regulated differentially expressed genes in Large White pigs than that in Mashen pigs.

Sequencing data for the 2 breeds were compared to determine the distribution of DEGs (Figure 1). Based on the screening criteria for DEGs (p<0.05, FDR<0.05, and |log2 (FC)|≤1), a total of 425 genes were differentially expressed between the breeds. Of these, 226 genes were upregulated in Large White pigs compared to Mashen pigs; 75 genes were known and 151 genes were unknown. Alternatively, 199 genes were upregulated in Mashen pigs compared to Large White pigs; 80 genes were known and 119 genes were unknown. The information of known DEGs were shown in Supplementary Table S1. When DEGs were further grouped based on FC in expression level, it was found that 108 genes in Large White pigs and 92 genes in Mashen pigs had a FC>100, while 33 genes in Large White pigs and 31 genes in Mashen pigs had a FC> 1,000 (Figure 2).

### Results of gene ontology and Kyoto encyclopedia of genes and genomes enrichment analyses

**Figure 3:**
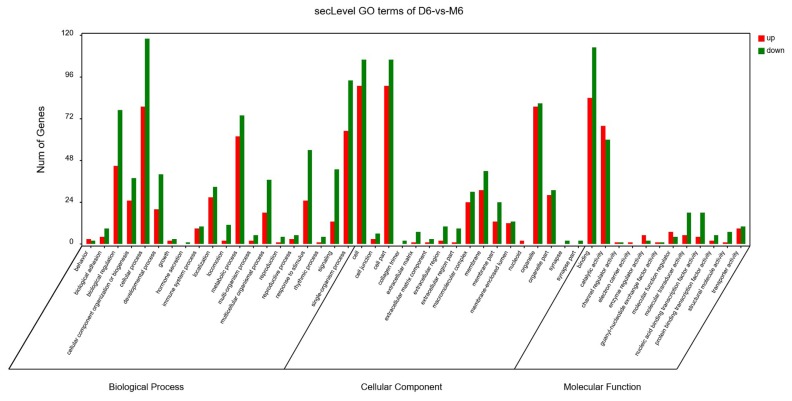
Gene ontology enrichment analysis of differentially expressed genes in Mashen and Large White pigs.

**Figure 4:**
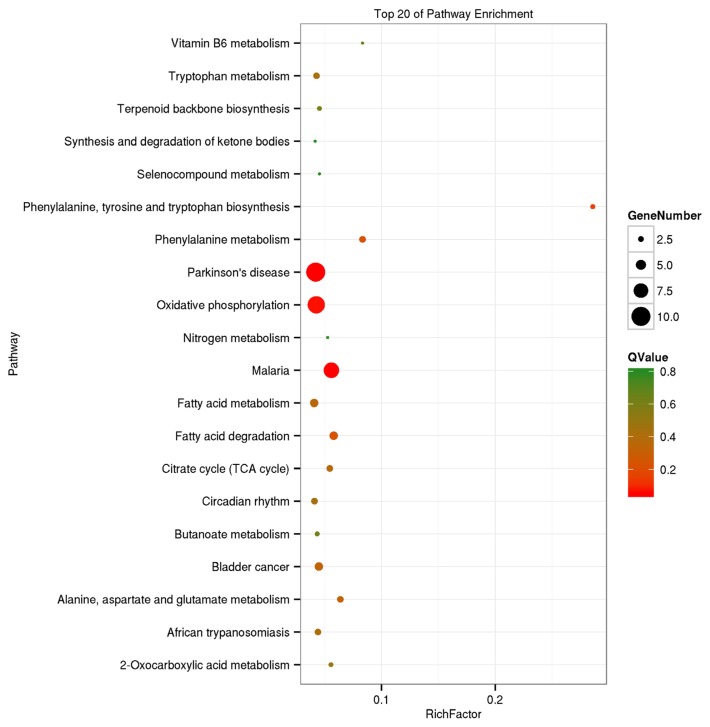
Kyoto encyclopedia of genes and genomes enrichment analysis of differentially expressed genes in Mashen and Large White pigs.

The GO analysis was used for the functional annotation and classification of DEGs. Genes are classified into three main groups: biological process (BP), cellular component (CC), and molecular function (MF). The results of our GO analysis (p<0.05; Figure 3) yielded a total of 50 significantly enriched terms among DEGs, including 21 BP terms, 17 CC terms, and 12 MF terms. The corresponding gene list was shown in Supplementary Table S2. The enriched terms potentially associated with meat quality were as follows: i) BP: metabolic processes, structural development processes, and regulation of gene expression; ii) CC: cell parts and mitochondria; iii) MF: redox activity, catalytic activity, and molecular binding. A KEGG analysis was used to facilitate an understanding of higher-order functions and biological systems at the molecular level. The top 20 pathways with significant enrichment among DEGs are shown in Figure 4 (p<0.05), and mainly included amino acid metabolism, oxidative phosphorylation, lipid metabolism and pathways associated with certain diseases (e.g., Parkinson’s disease and malaria). In particular, the expression levels of genes associated with oxidative phosphorylation were higher in Mashen pigs than in Large White pigs.

### Protein-protein interaction network of differentially expressed genes

**Figure 5:**
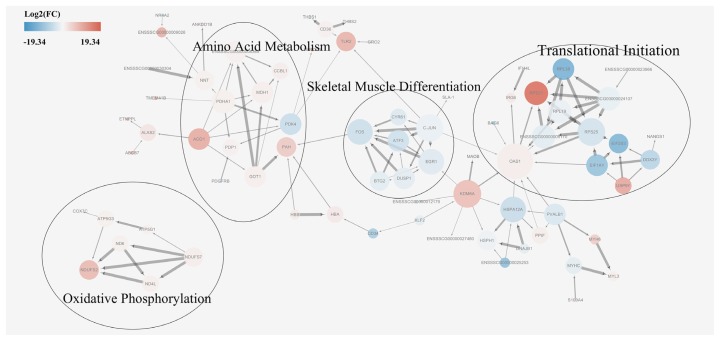
Protein-protein interaction network of differentially expressed genes. Red nodes indicate upregulation in Mashen pigs and blue nodes indicate upregulation in Large White pigs. Fold changes (FC) in expression are expressed as log2(FC) values. Node size indicates the extent to which the protein interacts with other proteins.

A protein-protein interaction network analysis was performed for DEGs in order to identify key candidate genes potentially affecting meat quality. First, a basic protein-protein interaction network was obtained from the STRING database. Then, a MCODE analysis was performed on the network using Cytoscape. Based on the MCODE algorithm, the network was divided into four subsets, each of which were subjected to a pathway enrichment analysis (Figure 5). The size of a single node in Figure 5 represents the number of proteins interacting with this single protein. The results showed that the enriched pathways among DEGs were translational initiation, skeletal muscle differentiation, amino acid metabolism, and oxidative phosphorylation. The module analysis results were consistent with that of the KEGG enrichment analysis results, supporting a possible relationship between proteins involved in skeletal muscle differentiation, amino acid metabolism, and oxidative phosphorylation pathways and meat quality. A combined analysis of the associated pathways and protein sizes in subsets led to the identification of the following core proteins: glutamic-oxaloacetic transaminase 1 (GOT1), malate dehydrogenase (MDH1), cytochrome c oxidase subunit 7C (COX 7c), and activator protein-1 (AP-1). It was hypothesized that these proteins had important effects on meat quality.

### Quantitative real-time polymerase chain reaction results

**Figure 6:**
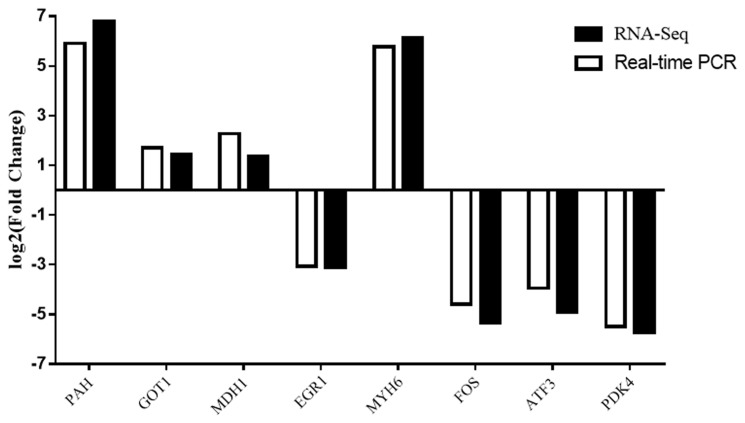
Comparison of quantitative real-time polymerase chain reaction and RNA-Seq results of differentially expressed genes.

To validate the reliability of the RNA-Seq results, we selected eight DEGs were for validation by qRT-PCR (Figure 6). The trends of gene expression were similar between qRT-PCR and RNA-Seq which indicated that the RNA-Seq results were reliable.

## DISCUSSION

The growth traits of pigs are mainly manifested in muscle growth and development, and the rate of muscle growth directly affects meat yield. Lean hogs such as the Large White have a significant advantage over Mashen pigs in terms of muscle growth rate. Skeletal muscle is an extremely heterogeneous tissue that is composed of a large variety of fiber types. Different types of skeletal muscle exhibit differences in metabolic (oxidative and glycolytic capacities), biochemical, and biophysical characteristics (fiber size, fiber color, glycogen content, and fat content) [15,16]. Muscle fibers are generally classified as type I or type II. Type I muscle fibers, also known as oxidative fibers, are rich in mitochondria and mainly produce energy through oxidative phosphorylation. Type II muscle fibers have a lower mitochondrial content and mainly produce ATP through the glycolytic pathway; these fibers can be further classified as type IIa, IIb, and IIx. Guo et al [17] examined differences in muscle fiber composition between 6-month-old Jinhua pigs (a Chinese domestic breed) and Landrace pigs (a western breed) and found that Jinhua pigs had significantly higher oxidative fiber content, especially type I muscle fibers, in the longissimus dorsi muscle tissue than Landrace pigs, while the content of Landrace pigs had higher glycolytic muscle fiber content, especially type IIb muscle fibers. In the present study, we found that gene expression levels of oxidative phosphorylation pathway components were higher in Mashen pigs than in Large White pigs, accounting for the significantly higher type I muscle fiber content and significantly lower type II muscle fiber content of skeletal muscle in Mashen pigs compared to Large White pigs. In addition to muscle fiber type, the number and diameter of muscle fibers are also key factors that affect muscle traits [18,19]. Myosin heavy chain 3 (MYH3) is a structural protein that affects muscle fiber type and its expression level is positively correlated with muscle fiber thickness. The expression level of *MYH3* is significantly higher in Large White pigs than in Mashen pigs, accounting for a higher growth rate and larger muscle fiber diameter in Large White pigs. The Gao study showed that muscle fiber diameter was significantly smaller and muscle fiber density was considerably higher in 180-day-old Mashen pigs than in Large White pigs [20]. Thinner and denser muscle fibers result in higher muscle fat content, which explains the observation of higher intramuscular fat content in Mashen pigs compared to Large White pigs to a certain extent. Furthermore, other studies have found that the expression level of myosin heavy chain 6 (*MYH6*) gene is significantly positively correlated with the density of type I muscle fibers, and sequencing results have indicated that *MYH6* gene expression is significantly higher in Mashen pigs compared to Large White pigs. Therefore, it can be hypothesized that the upregulation of *MYH6* expression in Mashen pigs results in a higher proportion of type I muscle fibers and smaller muscle fiber diameter.

The results of our MCODE analysis of the protein-protein interaction network revealed an association between the *AP-1* gene family and skeletal muscle differentiation. AP-1 is a transcription factor that regulates many BPs in cells including cell proliferation, differentiation, and apoptosis. AP-1 specifically serves as a transcription-activating molecular switch and regulates gene expression in response to cellular stress and other stimuli [21]. Members of the AP-1 family include c-Fos, c-Jun, and activating transcription factor (ATF); in particular, Fos forms homodimers or heterodimers with ATF and Jun to regulate gene expression [22], while ATF3 is involved in regulation of the cell cycle, apoptosis, and cellular responses to stress [23]. Studies have found that the induction of AP-1 increases the expression of vascular endothelial growth factor, which in turn promotes the proliferation of endothelial cells and affects angiogenesis [24]. Early growth response gene 1 (*Egr1*) promotes the expression of *AP-1*; the induction of *Egr1* in uterine leiomyoma cells led to a significant increase in the expression levels of Egr1, ATF3, Fos, and Jun [25]. AP-1 also participates in the oxidative stress response in skeletal muscle, and the oxidizing agent H_2_O_2_ induces AP-1 expression and exerts dose-dependent effects on the *AP-1* gene family [26]. The overexpression of members of the *AP-1* gene family accounts for a high muscle growth rate, large muscle fiber diameter, and low intramuscular fat content in Large White pigs.

Indicators of meat quality include color, tenderness, and moisture content, which are all closely related to muscle fiber type and intramuscular fat content. Additionally, amino acid metabolism exerts a key influence on meat quality. In the present study, DEGs were enriched in amino acid metabolism pathways include *GOT1*, *MDH1*, monocarboxylate transporter 4 (*SLC16A3*), pyruvate dehydrogenase 1 (*PDHA1*), and pyruvate dehydrogenase kinase 4 (*PDK4*).

**Figure 7:**
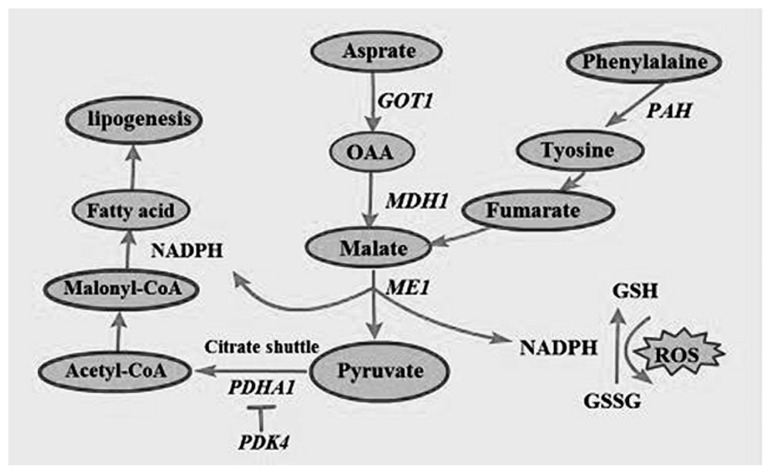
Fatty acid synthesis and antioxidant processes involving the participation of genes associated with amino acid metabolism.

GOT1 is a key enzyme that is involved in amino acid metabolism and carbohydrate metabolism and is mainly distributed in tissues such as the heart, liver, skeletal muscle, and kidneys [27]. In addition to regulating amino acid metabolism, GOT1 also promotes tumor cell proliferation by maintaining intracellular redox balance [28]. Previous studies have shown that GOT1 expression is closely linked to cell proliferation and glutamate synthesis. The SLC16A3 enables the transport of aspartate in the mitochondria to the cytoplasmic matrix, thus providing raw materials for the synthesis of proteins and other amino acids [29]; this is consistent with the assertion that Chinese domestic pig breeds have a higher content of flavor-producing amino acids such as aspartate and glutamate compared to breeds introduced from western countries [30]. The content of these flavor-producing amino acids and others that act as precursors to many flavor substances have been identified as an important reason for the sweetness of the meat of Chinese domestic pigs such as Mashen pigs [31]. During amino acid metabolism, GOT1 catalyzes the conversion of mitochondrial aspartate to oxaloacetate (OAA) in the cytoplasmic matrix, while MDH1 catalyzes the conversion of OAA to malate. Subsequently, malic enzyme 1 (ME1) catalyzes the conversion of malate to Nicotinamide adenine dinucleotide phosphate (NADPH), which has antioxidant effects and can reduce glutathione disulfide (GSSG) to glutathione (GSG). GSG neutralizes intracellular H2O2 to protect cells from oxidative stress [32,33], while the overexpression of MDH1 leads to an increase in NADPH production [34]. A previous study found that MDH1 activity was higher in Mashen pigs than in Large White pigs [10]. Another study by Zeng et al [35] revealed that *MDH1* expression in the muscle tissue of Laiwu Black pigs was significantly positively correlated with intramuscular fat content. Kim et al [36] overexpressed *MDH1* in 3T3-L1 cells with an retroviral infection system (IRES-GFP) vector and differentiated infected cells into mature adipocytes; as a result, it was found that MDH1 overexpression significantly increased lipid accumulation and was associated with the increased expression of lipogenesis markers such as PPARγ and C/EBPα. These findings indicated that MDH1 influences meat quality by affecting intramuscular fat content. In addition to maintaining intracellular redox balance, malate produced in the aforementioned process is also converted to pyruvate by ME1. Subsequently, pyruvate enters the citric acid cycle and is converted to acetyl-CoA, H+, CO_2_, and NADH by pyruvate dehydrogenase complex (PDHC)-catalyzed oxidative decarboxylation. E1 (pyruvate dehydrogenase), a core component of PDHC, is composed of 2 α-subunits encoded by the *PDHA1* gene and located in the mitochondrial matrix. The main product of the PDHA1-catalyzed reaction, Acetyl-CoA, is an important substrate of the citric acid cycle, fatty acid synthesis, and cholesterol synthesis. The PDK and pyruvate dehydrogenase phosphatase participate in the phosphorylation and dephosphorylation of PDHA1 [37]. PDHA1 mutations can produce disorders of mitochondrial function and have been implicated in disease states such as epilepsy and Alzheimer’s disease [38]. This explains significant enrichment observed for brain-related diseases such as Parkinson’s disease in our pathway enrichment analysis results. NADH transfers electrons to oxygen via cytochrome c oxidase (COX 7c) and contributes to the formation of a proton gradient that drives ATP synthesis in the mitochondrial intermembrane space, a process that accounts for the synthesis of >94% of ATP in organisms [39]. PDK4 plays a vital role in the regulation of glucose and fatty acid metabolism and also exerts a major influence on cell proliferation by regulating carbohydrate and fatty acid metabolism. PDK4 inhibits pyruvate dehydrogenase activity to decrease aerobic respiration and inhibit the formation of acetyl-CoA, thereby promoting fatty acid metabolism. PDHA1 activity increases in the presence of dichloroacetic acid, which is a PDK inhibitor [40]. A summary of these fatty acid synthesis processes and participating proteins associated with amino acid metabolism are shown in Figure 7.

## CONCLUSION

In the present study, RNA-Seq was used to perform transcriptome sequencing of longissimus dorsi muscle tissues from Mashen pigs and Large White pigs. A subsequent analysis identified 425 DEGs, with 226 upregulated genes and 199 downregulated genes in Large White pigs compared to Mashen pigs. GO, KEGG, and protein-protein interaction analyses identified *GOT1*, *MDH1*, *PDHA1*, *PDK4*, and *AP-1* as candidate genes affecting meat quality traits, while amino acid metabolism, oxidative phosphorylation, and regulation of gene expression were identified as metabolic pathways and BPs significantly associated with meat quality traits. Our results provide a preliminary understanding of the reasons for differences in meat quality traits between Mashen pigs and Large White pigs, and provide a theoretical basis for studies on candidate genes and the improvement of meat quality in pigs.

## Supplementary Data



## Figures and Tables

**Table 1:** Primer sequences of eight differentially expressed genes and *18S rRNA* used in quantitative real-time polymerase chain reaction

Gene	Primer sequence (5′-3′)	Product length (bp)
*PAH*	F: GTACATCAGACACGCGTCCA	114
	R: TCCTGGGAAAACTGGGCAAA	
*GOT1*	F: CATCCTGCGAGTCCTTTC	150
	R: CGGTCAGCCATTGTCTTC	
*MDH1*	F: TAAGGTTATCGTGGTGGG	124
	R: TGCTTTAGCTCGGTTGTG	
*EGR1*	F: TTCCCTTTCCTCCGCAGTTC	146
	R: GGGTCAGGCATACGATGGAG	
*MYH6*	F: CTGACCAGGTGACCCCTAAC	116
	R: CCACAGGGCATATTTGGACC	
*FOS*	F: GGGAGCTGACTGACACACTC	125
	R: GTGAGCTGCCAGGATGAACT	
*ATF3*	F: TGGAGACAGGAGCAAAATGAT	110
	R: CAAACACCAGTGACCCAGGA	
*PDK4*	F: CCAGGATATGGAACGGATGC	141
	R: GCTGCTTTCTTCGCCAACC	
*18S rRNA*	F: CCCACGGAATCGAGAAAGAG	122
	R: TTGACGGAAGGGCACCA	

*PAH*, phenylalanine hydroxylase; *GOT1*, glutamic-oxaloacetic transaminase 1; *MDH1*, malate dehydrogenase 1; *EGR1*, early growth response gene 1; *MYH6*, myosin heavy chain 6; *FOS*, fos proto-oncogene; *ATF3*, activating transcription factor 3; *PDK4*, pyruvate dehydrogenase kinase 4.

**Table 2:** Number of RNA-Seq reads and Q value

Sample	Raw bases (G)	Clean reads (G)	Q20 (%)	Q30 (%)
L61	17.4	16.5	95.9	86.7
L62	16.6	15.6	95.9	86.4
L63	19.4	18.3	95.9	86.7
M61	14.2	13.5	96.0	87.1
M62	13.7	13.0	96.1	87.5
M63	17.4	16.5	96.2	87.6

L, White pig; M, Mashen pig.

**Table 3:** Number of RNA-seq reads and mapping results1)

Samples	Total reads (bp)	Unmapped reads (bp)	Unique mapped reads (bp)	Multiple mapped reads (bp)	Mapping ratio (%)
L61	169,547,196	43,292,454 (25.5%)	123,384,970 (72.8%)	2,869,772 (1.69%)	74.5
L62	161,379,056	69,499,637 (43.1%)	89,810,577 (55.7%)	2,068,842 (1.28%)	56.9
L63	188,702,130	66,528,342 (35.3%)	119,154,238 (63.1%)	3,019,550 (1.60%)	64.7
M61	138,403,716	35,983,885 (26.0%)	100,380,419 (72.5%)	2,039,412 (1.47%)	74.0
M62	133,386,674	33,612,816 (25.2%)	97,700,986 (73.6%)	2,072,872 (1.55%)	74.8
M63	169,553,386	44,019,176 (26.0%)	121,851,714 (71.9%)	3,682,496 (2.17%)	74.0
Total	960,972,158	292,936,310 (30.5%)	652,282,904 (67.9%)	15,752,944 (1.65%)	69.5

L, White pig; M, Mashen pig.

1) The percentage of each column in the table represents the ratio of the column to the total logarithmic ratio.
