# Supplementary material for: Selection of candidate genes affecting meat quality and preliminary exploration of related molecular mechanisms in the Mashen pig
Source: Asian-Australas J Anim Sci. 2019 Feb 14;32(8):1084–94. doi: 10.5713/ajas.18.0718 (PMC6599955; doi:10.5713/ajas.18.0718)
Supplement: Supplementary file 1 [file ajas-18-0718-suppl.pdf]

Table S1 The information of known DEGs

id	D6_fpkm	M6_fpkm	log2(FC)	Symbol	Description
ENSSSCT00000000144	5.41	0.113333	-5.57698	PVALB	parvalbumin [Source:HGNC Symbol;Acc:HGNC:9704]
ENSSSCT00000000392	0.001	1.236667	10.27224	PYM1	PYM homolog 1, exon junction complex associated factor [Source:HGNC Symbol;Acc:HGNC:30258]
ENSSSCT00000000934	0.016667	1.836667	6.78398	PAH	phenylalanine hydroxylase [Source:HGNC Symbol;Acc:HGNC:8582]
ENSSSCT00000001193	0.001	0.253333	7.984893	FAM65B	family with sequence similarity 65, member B [Source:HGNC Symbol;Acc:HGNC:13872]
ENSSSCT00000001329	56.90333	125.35	1.139377	TMP-CH242-74M17.2	-
ENSSSCT00000001927	7.633333	20.51333	1.426177	PLA2G7	phospholipase A2, group VII (platelet-activating factor acetylhydrolase, plasma) [Source:HGNC Symbol;Acc:HGNC:9040]
ENSSSCT00000002252	15.03	44.52667	1.566825	DHRS4	Dehydrogenase/reductase SDR family member 4 [Source:UniProtKB/Swiss-Prot;Acc:Q8WNV7]
ENSSSCT00000002510	0.83	0.003333	-7.96	NR2F2	nuclear receptor subfamily 2, group F, member 2 [Source:HGNC Symbol;Acc:HGNC:7976]
ENSSSCT00000002650	97.94	2.403333	-5.34879	FOS	-
ENSSSCT00000002713	0.243333	0.001	-7.92679	TC2N	tandem C2 domains, nuclear [Source:HGNC Symbol;Acc:HGNC:19859]
ENSSSCT00000002808	0.001	664.8433	19.34265	-	40S ribosomal protein S21 [Source:UniProtKB/Swiss-Prot;Acc:P63221]
ENSSSCT00000002812	0.03	2.223333	6.211618	ZNF839	zinc finger protein 839 [Source:HGNC Symbol;Acc:HGNC:20345]
ENSSSCT00000003504	21.79	5.06	-2.10646	PPP1R15A	protein phosphatase 1, regulatory subunit 15A

					[Source:HGNC Symbol;Acc:HGNC:14375]
ENSSSCT00000003939	83.85667	367.88	2.133238	-	-
ENSSSCT00000004174	0.001	1.026667	10.00375	ZZZ3	zinc finger, ZZ-type containing 3 [Source:HGNC Symbol;Acc:HGNC:24523]
ENSSSCT00000004436	5.663333	1.893333	-1.58072	THBS2	thrombospondin 2 [Source:HGNC Symbol;Acc:HGNC:11786]
ENSSSCT00000005271	0.77	0.001	-9.58871	BAHD1	bromo adjacent homology domain containing 1 [Source:HGNC Symbol;Acc:HGNC:29153]
ENSSSCT00000005288	15.11333	4.053333	-1.89864	THBS1	-
ENSSSCT00000005633	0.13	0.001	-7.02237	SYNE2	spectrin repeat containing, nuclear envelope 2 [Source:HGNC Symbol;Acc:HGNC:17084]
ENSSSCT00000005967	16.36333	55.52667	1.762714	NIPSNAP3B	nipsnap homolog 3B (C. elegans) [Source:HGNC Symbol;Acc:HGNC:23641]
ENSSSCT00000006226	3.243333	10.48	1.69209	CCBL1	cysteine conjugate-beta lyase, cytoplasmic [Source:HGNC Symbol;Acc:HGNC:1564]
ENSSSCT00000006644	27.02333	9.63	-1.4886	KLF10	-
ENSSSCT00000006699	16.11667	36.75	1.189191	PDP1	pyruvate dehyrogenase phosphatase catalytic subunit 1 [Source:HGNC Symbol;Acc:HGNC:9279]
ENSSSCT00000006706	0.001	1.856667	10.8585	CU207250.1	-
ENSSSCT00000006733	7.126667	2.496667	-1.51322	CA2	carbonic anhydrase II [Source:HGNC Symbol;Acc:HGNC:1373]
ENSSSCT00000006967	0.001	0.556667	9.12067	NDUFS2	NADH dehydrogenase (ubiquinone) Fe-S protein 2, 49kDa (NADH-coenzyme Q reductase) [Source:HGNC Symbol;Acc:HGNC:7708]
ENSSSCT00000007206	0.403333	5.16	3.677327	S100A4	S100 calcium binding protein A4 [Source:HGNC Symbol;Acc:HGNC:10494]
ENSSSCT00000007534	0.001	1.286667	10.32942	-	-
ENSSSCT00000007573	0.001	0.353333	8.464886	-	-

ENSSSCT00000007604	7.406667	0.753333	-3.29746	CYR61	cysteine-rich, angiogenic inducer, 61 [Source:HGNC Symbol;Acc:HGNC:2654]
ENSSSCT00000007834	0.936667	0.001	-9.87139	-	-
ENSSSCT00000008247	0.756667	0.001	-9.56351	GNAS	Sus scrofa GNAS complex locus (GNAS), transcript variant 1, mRNA. [Source:RefSeq mRNA;Acc:NM_214312]
ENSSSCT00000008741	49.60667	227.2367	2.19559	HBQ1	hemoglobin, theta 1 [Source:HGNC Symbol;Acc:HGNC:4833]
ENSSSCT00000008947	9.223333	1.52	-2.60122	-	60S ribosomal protein L31 [Source:UniProtKB/Swiss-Prot;Acc:P62901]
ENSSSCT00000009163	101.1733	243.73	1.268455	-	-
ENSSSCT00000009164	68.20333	174.8767	1.358424	-	-
ENSSSCT00000009339	0.466667	5.57	3.577213	CLIP4	CAP-GLY domain containing linker protein family, member 4 [Source:HGNC Symbol;Acc:HGNC:26108]
ENSSSCT00000009375	50.39	124.28	1.302385	HADHB	long-chain 3-ketoacyl-CoA thiolase [Source:RefSeq peptide;Acc:NP_999131]
ENSSSCT00000009461	1.64	7.583333	2.209136	RSAD2	-
ENSSSCT00000009813	4.433333	0.153333	-4.85365	CXCL2	C-X-C motif chemokine 2 precursor [Source:RefSeq peptide;Acc:NP_001001861]
ENSSSCT00000009859	0.001	1.023333	9.99906	TLR2	toll-like receptor 2 [Source:HGNC Symbol;Acc:HGNC:11848]
ENSSSCT00000009885	0.001	1.533333	10.58246	-	-
ENSSSCT00000010013	0.003333	0.92	8.108524	ETNPPL	ethanolamine-phosphate phospho-lyase [Source:HGNC Symbol;Acc:HGNC:14404]
ENSSSCT00000010151	1.303333	0.001	-10.348	-	-
ENSSSCT00000010229	19.25667	8.03	-1.26189	HSPH1	heat shock protein 105 kDa [Source:RefSeq peptide;Acc:NP_001090973]
ENSSSCT00000010558	0.001	6.673333	12.70419	PEBP4	Sus scrofa phosphatidylethanolamine-binding protein 4 (PEBP4),

					mRNA. [Source:RefSeq mRNA;Acc:NM_001162888]
ENSSSCT00000010832	3.103333	9.783333	1.656507	OAS1	2'-5'-oligoadenylate synthetase 1, 40/46kDa [Source:HGNC Symbol;Acc:HGNC:8086]
ENSSSCT00000011053	0.25	0.001	-7.96578	PI4KA	phosphatidylinositol 4-kinase, catalytic, alpha [Source:HGNC Symbol;Acc:HGNC:8983]
ENSSSCT00000011308	5.313333	16.15333	1.604143	-	-
ENSSSCT00000011527	63.87333	173.52	1.441816	GOT1	Aspartate aminotransferase, cytoplasmic [Source:UniProtKB/Swiss-Prot;Acc:P00503]
ENSSSCT00000011662	0.84	0.013333	-5.97728	HSPA12A	heat shock 70kDa protein 12A [Source:HGNC Symbol;Acc:HGNC:19022]
ENSSSCT00000011679	4.763333	0.886667	-2.42551	NANOS1	nanos homolog 1 (Drosophila) [Source:HGNC Symbol;Acc:HGNC:23044]
ENSSSCT00000012400	378.2933	994.8167	1.394925	MYL3	-
ENSSSCT00000012465	0.001	0.646667	9.336878	-	-
ENSSSCT00000013322	19.83	0.001	-14.2754	-	-
ENSSSCT00000013323	5.7	0.086667	-6.03934	-	-
ENSSSCT00000013502	0.52	3.57	2.779341	ALAS2	5'-aminolevulinate synthase 2 [Source:HGNC Symbol;Acc:HGNC:397]
ENSSSCT00000013571	1.623333	12.83	2.98249	PHKA1	phosphorylase kinase, alpha 1 (muscle) [Source:HGNC Symbol;Acc:HGNC:8925]
ENSSSCT00000014241	4.983333	14.59333	1.550126	ESRRA	estrogen-related receptor alpha [Source:HGNC Symbol;Acc:HGNC:3471]
ENSSSCT00000014538	0.001	2.046667	10.99906	HIPK3	homeodomain interacting protein kinase 3 [Source:HGNC Symbol;Acc:HGNC:4915]
ENSSSCT00000014800	0.001	1.086667	10.08569	-	-
ENSSSCT00000015061	94.59333	17.26	-2.45431	-	-
ENSSSCT00000015384	0.23	7.33	4.994107	ANKDD1B	ankyrin repeat and death domain containing 1B [Source:HGNC Symbol;Acc:HGNC:32525]

ENSSSCT00000015432	355.4867	841.37	1.242945	CKMT2	-
ENSSSCT00000015449	11.43667	29.9	1.386479	-	Cytochrome c oxidase subunit 7C, mitochondrial [Source:UniProtKB/Swiss-Prot;Acc:Q1W0Y2]
ENSSSCT00000015576	53.64667	19.52333	-1.45829	SLC12A2	solute carrier family 12 (sodium/potassium/chloride transporter), member 2 [Source:HGNC Symbol;Acc:HGNC:10911]
ENSSSCT00000015660	7.37	0.846667	-3.1218	EGR1	early growth response 1 [Source:HGNC Symbol;Acc:HGNC:3238]
ENSSSCT00000015663	1.56	0.01	-7.2854	EGR1	early growth response 1 [Source:HGNC Symbol;Acc:HGNC:3238]
ENSSSCT00000015929	0.001	1.066667	10.05889	-	-
ENSSSCT00000016196	1.433333	0.001	-10.4852	-	-
ENSSSCT00000016469	80.38	3.313333	-4.60048	RPS25	ribosomal protein S25 [Source:HGNC Symbol;Acc:HGNC:10413]
ENSSSCT00000016707	421.04	7.9	-5.73596	PDK4	-
ENSSSCT00000016780	15.67333	37.79667	1.269947	CD36	-
ENSSSCT00000016961	13.36333	44.67333	1.741134	IVNS1ABP	influenza virus NS1A binding protein [Source:HGNC Symbol;Acc:HGNC:16951]
ENSSSCT00000017016	0.786667	0.001	-9.61961	CD34	CD34 molecule [Source:HGNC Symbol;Acc:HGNC:1662]
ENSSSCT00000017270	1.006667	0.001	-9.97537	SAP130	Sin3A-associated protein, 130kDa [Source:HGNC Symbol;Acc:HGNC:29813]
ENSSSCT00000017282	2.286667	0.086667	-4.72163	NR4A2	nuclear receptor subfamily 4, group A, member 2 [Source:HGNC Symbol;Acc:HGNC:7981]
ENSSSCT00000017392	66.26667	167.0867	1.334241	ATP5G3	-
ENSSSCT00000017523	33.59	10.87	-1.62768	-	-
ENSSSCT00000018009	43.86	147.4	1.748759	AKR1B1	-
ENSSSCT00000018236	9	2.916667	-1.6256	AEBP1	AE binding protein 1 [Source:HGNC Symbol;Acc:HGNC:303]

ENSSSCT00000018381	56.09667	129.4033	1.205888	NNT	nicotinamide nucleotide transhydrogenase [Source:HGNC Symbol;Acc:HGNC:7863]
ENSSSCT00000018501	130.4567	25.56667	-2.35123	DUSP1	-
ENSSSCT00000018768	38.74	0.001	-15.2415	RPL38	ribosomal protein L38 [Source:HGNC Symbol;Acc:HGNC:10349]
ENSSSCT00000019062	69.26	22.72	-1.60806	-	-
ENSSSCT00000019098	219.1333	526.94	1.26583	ATP5G1	ATP synthase, H+ transporting, mitochondrial Fo complex, subunit C1 (subunit 9) [Source:HGNC Symbol;Acc:HGNC:841]
ENSSSCT00000019601	6.24	1.123333	-2.47376	-	-
ENSSSCT00000019681	360.45	987.2767	1.453655	ND4L	NADH dehydrogenase subunit 4L (mitochondrion) [Source:RefSeq peptide;Acc:NP_008642]
ENSSSCT00000019687	448.2767	1029.107	1.198931	ND6	NADH-ubiquinone oxidoreductase chain 6 [Source:UniProtKB/Swiss-Prot;Acc:O79882]
ENSSSCT00000022502	0.001	0.46	8.84549	-	-
ENSSSCT00000023318	33.22	108.5567	1.708324	NDUFS7	NADH dehydrogenase (ubiquinone) Fe-S protein 7, 20kDa (NADH-coenzyme Q reductase) [Source:HGNC Symbol;Acc:HGNC:7714]
ENSSSCT00000023380	20.19333	46.93	1.216631	-	-
ENSSSCT00000023398	8.566667	0.08	-6.74259	-	-
ENSSSCT00000023699	0.001	2.043333	10.99671	ACO1	aconitase 1, soluble [Source:HGNC Symbol;Acc:HGNC:117]
ENSSSCT00000024200	1.493333	11.39333	2.931582	-	-
ENSSSCT00000024553	8.316667	0.001	-13.0218	-	-
ENSSSCT00000024741	0.536667	0.001	-9.06788	PPIL4	peptidylprolyl isomerase (cyclophilin)-like 4 [Source:HGNC Symbol;Acc:HGNC:15702]
ENSSSCT00000024980	30.77333	0.01	-11.5875	-	-
ENSSSCT00000025191	0.001	3.6	11.81378	MICU2	mitochondrial calcium uptake 2 [Source:HGNC Symbol;Acc:HGNC:31830]

ENSSSCT00000025648	0.493333	0.001	-8.94642	ARAP1	ArfGAP with RhoGAP domain, ankyrin repeat and PH domain 1 [Source:HGNC Symbol;Acc:HGNC:16925]
ENSSSCT00000026377	6.593333	1.346667	-2.29162	RASGEF1B	ras-GEF domain-containing family member 1B [Source:RefSeq peptide;Acc:NP_001182296]
ENSSSCT00000026451	14.86333	55.65667	1.904797	PPP1R3B	protein phosphatase 1, regulatory subunit 3B [Source:HGNC Symbol;Acc:HGNC:14942]
ENSSSCT00000026880	0.001	26.21	14.67783	-	-
ENSSSCT00000027232	188.0133	830.4833	2.143116	-	-
ENSSSCT00000027235	0.74	0.001	-9.53138	RFFL	ring finger and FYVE-like domain containing E3 ubiquitin protein ligase [Source:HGNC Symbol;Acc:HGNC:24821]
ENSSSCT00000027298	12.68	4.206667	-1.59181	-	-
ENSSSCT00000027462	0.383333	0.006667	-5.84549	WDR19	WD repeat domain 19 [Source:HGNC Symbol;Acc:HGNC:18340]
ENSSSCT00000027771	121.9833	308.7767	1.33988	PDHA1	pyruvate dehydrogenase (lipoamide) alpha 1 [Source:HGNC Symbol;Acc:HGNC:8806]
ENSSSCT00000027869	6.656667	1.436667	-2.21207	-	-
ENSSSCT00000028216	224.77	64.15667	-1.80878	-	-
ENSSSCT00000028237	45.18333	110.9167	1.295614	-	-
ENSSSCT00000028542	6.95	1.233333	-2.49445	-	-
ENSSSCT00000028661	102.8833	17.34667	-2.56828	BTG2	-
ENSSSCT00000028884	0.193333	0.001	-7.59495	DDX3Y	DEAD (Asp-Glu-Ala-Asp) box helicase 3, Y-linked [Source:HGNC Symbol;Acc:HGNC:2699]
ENSSSCT00000028975	110.8933	20.70667	-2.42101	SHISA2	shisa family member 2 [Source:HGNC Symbol;Acc:HGNC:20366]
ENSSSCT00000029040	10.93667	0.001	-13.4169	-	-
ENSSSCT00000029270	4.463333	12	1.426841	IFI44L	interferon-induced protein 44-like [Source:HGNC Symbol;Acc:HGNC:17817]

ENSSSCT00000029625	0.001	3.1	11.59805	USP9Y	ubiquitin specific peptidase 9, Y-linked [Source:HGNC Symbol;Acc:HGNC:12633]
ENSSSCT00000029905	0.001	1.39	10.44087	FKBP9	FK506 binding protein 9, 63 kDa [Source:HGNC Symbol;Acc:HGNC:3725]
ENSSSCT00000030563	0.056667	3.093333	5.770518	-	-
ENSSSCT00000030802	0.743333	0.001	-9.53787	-	-
ENSSSCT00000030852	2.4	0.001	-11.2288	-	-
ENSSSCT00000030903	5.53	17.15333	1.633138	-	-
ENSSSCT00000030906	0.12	0.001	-6.90689	KIAA2026	KIAA2026 [Source:HGNC Symbol;Acc:HGNC:23378]
ENSSSCT00000031037	0.001	0.41	8.67948	ETV6	ets variant 6 [Source:HGNC Symbol;Acc:HGNC:3495]
ENSSSCT00000031092	0.001	0.92	9.84549	-	-
ENSSSCT00000031122	0.001	0.96	9.906891	P4HA2	prolyl 4-hydroxylase, alpha polypeptide II [Source:HGNC Symbol;Acc:HGNC:8547]
ENSSSCT00000031202	6.266667	0.001	-12.6135	-	-
ENSSSCT00000031428	1.976667	6.473333	1.711439	SLC16A3	solute carrier family 16 (monocarboxylate transporter), member 3 [Source:HGNC Symbol;Acc:HGNC:10924]
ENSSSCT00000031509	3.393333	1.16	-1.54858	-	-
ENSSSCT00000031950	5.616667	0.38	-3.88564	ADAMTS1	-
ENSSSCT00000031972	2.696667	33.12667	3.618744	DEFB1	-
ENSSSCT00000032277	0.246667	0.001	-7.94642	UBR1	ubiquitin protein ligase E3 component n-recognin 1 [Source:HGNC Symbol;Acc:HGNC:16808]
ENSSSCT00000032593	11.29667	69.40333	2.619108	CD36	-
ENSSSCT00000032649	2.423333	0.443333	-2.45053	KLF4	Kruppel-like factor 4 (gut) [Source:HGNC Symbol;Acc:HGNC:6348]
ENSSSCT00000032662	0.001	3.06	11.57932	LIFR	leukemia inhibitory factor receptor alpha [Source:HGNC Symbol;Acc:HGNC:6597]
ENSSSCT00000033729	0.816667	0.001	-9.6736	TLR2	toll-like receptor 2 [Source:HGNC Symbol;Acc:HGNC:11848]

ENSSSCT00000033745	0.433333	3.166667	2.869416	MAOB	monoamine oxidase B [Source:HGNC Symbol;Acc:HGNC:6834]
ENSSSCT00000034484	0.826667	0.001	-9.69116	ACVR2B	activin A receptor, type IIB [Source:HGNC Symbol;Acc:HGNC:174]
ENSSSCT00000034640	9.07	1.403333	-2.69224	CXCL2	C-X-C motif chemokine 2 precursor [Source:RefSeq peptide;Acc:NP_001001861]
ENSSSCT00000034919	0.001	0.32	8.321928	KDM6A	lysine (K)-specific demethylase 6A [Source:HGNC Symbol;Acc:HGNC:12637]
ENSSSCT00000035032	1.276667	0.001	-10.3182	SMS	spermine synthase [Source:HGNC Symbol;Acc:HGNC:11123]
ENSSSCT00000035069	0.536667	37.23333	6.116425	MYH6	-
ENSSSCT00000035292	1.81	0.001	-10.8218	SCD	-
ENSSSCT00000035485	20.63333	0.683333	-4.91624	ATF3	activating transcription factor 3 [Source:HGNC Symbol;Acc:HGNC:785]
ENSSSCT00000035749	2.06	0.001	-11.0084	FADD	protein FADD [Source:RefSeq peptide;Acc:NP_001026967]
ENSSSCT00000035876	123.0433	9.196667	-3.74191	PVALB	parvalbumin [Source:HGNC Symbol;Acc:HGNC:9704]
ENSSSCT00000035973	1.206667	0.001	-10.2368	BAG6	BCL2-associated athanogene 6 [Source:HGNC Symbol;Acc:HGNC:13919]
ENSSSCT00000035982	2.516667	0.1	-4.65344	CH242-138G12.1	-
ENSSSCT00000036290	73.12	23.34667	-1.64705	JUN	-
ENSSSCT00000036352	0.843333	37.77333	5.485121	MYH6	-
ENSSSCT00000036373	4.123333	0.06	-6.1027	TSPAN6	tetraspanin-6 [Source:RefSeq peptide;Acc:NP_001230990]
ENSSSCT00000036434	25.99333	58.51	1.170542	PDHA1	pyruvate dehydrogenase (lipoamide) alpha 1 [Source:HGNC Symbol;Acc:HGNC:8806]
ENSSSCT00000036440	0.001	1.2	10.22882	ABCB7	ATP-binding cassette, sub-family B (MDR/TAP), member 7 [Source:HGNC Symbol;Acc:HGNC:48]

ENSSSCT00000036536	121.4167	455.2533	1.906703	HBB	-

Table S2 The corresponding gene list of GO analysis

Ontology	Class	number_of_D6-vs-M6_up	number_of_D6-vs-M6_down	genes_of_D6-vs-M6_up	genes_of_D6-vs-M6_down
Biological Process	metabolic process	62	74	ENSSSCT00000000934/ENSSSCT00000001927/ENSSSCT00000002808/ENSSSCT00000006226/ENSSSCT00000006699/ENSSSCT00000007534/ENSSSCT00000009163/ENSSSCT00000009164/ENSSSCT00000011308/ENSSSCT00000011527/ENSSSCT00000013502/ENSSSCT00000013571/ENSSSCT00000014241/ENSSSCT00000014538/ENSSSCT00000017392/ENSSSCT00000018381/ENSSSCT00000019098/ENSSSCT00000019681/ENSSSCT00000026451/ENSSSCT00000027771/ENSSSCT00000029625/ENSSSCT00000029905/ENSSSCT00000031122/ENSSSCT00000034919/ENSSSCT00000036434/TCONS_00001139/TCONS_00004794/TCONS_00004931/TCONS_00007403/TCONS_00013396/TCONS_00014448/TCONS_00016859/TCONS_00018210/TCONS_00020459/TCONS_00024355/TCONS_00028863/TCONS_00032517/TCONS_00033609/TCONS_000	ENSSSCT00000002510/ENSSSCT00000006733/ENSSSCT00000008947/ENSSSCT00000011053/ENSSSCT00000013322/ENSSSCT00000015061/ENSSSCT00000017282/ENSSSCT00000017523/ENSSSCT00000018501/ENSSSCT00000018768/ENSSSCT00000019062/ENSSSCT00000024741/ENSSSCT00000024980/ENSSSCT00000027462/ENSSSCT00000028216/ENSSSCT00000028884/ENSSSCT00000031202/ENSSSCT00000032277/ENSSSCT00000034484/ENSSSCT00000035032/ENSSSCT00000035292/ENSSSCT00000035973/ENSSSCT00000035982/ENSSSCT00000036290/TCONS_00004796/TCONS_00005241/TCONS_00006314/TCONS_00006316/TCONS_00008348/TCONS_00011158/TCONS_00011474/TCONS_00012424/TCONS_00013318/TCONS_00013451/TCONS_00013452/TCONS_00015685/TCONS_00016536/TCONS_00017991/TCONS_00020520/TCONS_00022007/TCONS_00028230/TCONS_00029348/TCONS_00032031/TCONS_00035093/TCONS_00038058/TCONS_00041695/TCONS_00042856/TCONS_00045347/

				36842/TCONS_00042642/TCONS_00045665/TCONS_00046597/TCONS_00049007/TCONS_00051555/TCONS_00052894/TCONS_00055773/TCONS_00059949/TCONS_00064907/TCONS_00065482/TCONS_00067733/TCONS_00075802/TCONS_00076786/TCONS_00080151/TCONS_00081239/TCONS_00083694/TCONS_00087175/TCONS_00088728/TCONS_00090200/TCONS_00090930/TCONS_00096376/TCONS_00098418/TCONS_00099248	TCONS_00045349/TCONS_00045630/TCONS_00048384/TCONS_00049436/TCONS_00052677/TCONS_00052953/TCONS_00068370/TCONS_00076154/TCONS_00076744/TCONS_00078029/TCONS_00081205/TCONS_00083246/TCONS_00084390/TCONS_00084498/TCONS_00085534/TCONS_00085544/TCONS_00092241/TCONS_00094370/TCONS_00098419/TCONS_00100494/TCONS_00100510/TCONS_00100513/TCONS_00100515/TCONS_00100528/TCONS_00100529/TCONS_00100533
Biological Process	rhythmic process	1	4	TCONS_00095452	TCONS_00016536/TCONS_00020520/TCONS_00048384/TCONS_00085534
Biological Process	developmental process	20	40	ENSSSCT00000003939/ENSSSCT00000027232/ENSSSCT00000031037/TCONS_00004931/TCONS_00010673/TCONS_00012908/TCONS_00016637/TCONS_00020459/TCONS_00033609/TCONS_00042642/TCONS_00049007/TCONS_00050395/TCONS_00051311/TCONS_00055773/TCONS_00057499/TCONS_00072374/TCONS_00090200/TCONS_00090930/TCONS_00095452/TCONS_00096376	ENSSSCT00000010229/ENSSSCT00000018501/ENSSSCT00000027462/ENSSSCT00000035973/TCONS_00003027/TCONS_00004822/TCONS_00006314/TCONS_00006316/TCONS_00006377/TCONS_00007428/TCONS_00008709/TCONS_00011158/TCONS_00016869/TCONS_00016870/TCONS_00017991/TCONS_00024980/TCONS_00024981/TCONS_00028230/TCONS_00031135/TCONS_00031548/TCONS_00035093/TCONS_00035601/TCONS_00038058/TCONS_00045630/TCONS_00045691/TCONS_00045692/TCONS_00049436/TCONS_00050770/TCONS_00052642/TCONS_00053886/TCONS_0

					0054717/TCONS_00058416/TCONS_00081205/TCONS_00084498/TCONS_00085534/TCONS_00085544/TCONS_00092241/TCONS_00096230/TCONS_00100528/TCONS_00100529
Biological Process	cellular component organization or biogenesis	25	38	ENSSSCT00000002808/ENSSSCT00000011308/ENSSSCT00000034919/TCONS_00000968/TCONS_00004931/TCONS_00009137/TCONS_00016637/TCONS_00025216/TCONS_00028863/TCONS_00032517/TCONS_00033609/TCONS_00042642/TCONS_00046597/TCONS_00049538/TCONS_00050395/TCONS_00051311/TCONS_00051555/TCONS_00055773/TCONS_00062759/TCONS_00067733/TCONS_00087195/TCONS_00090200/TCONS_00094383/TCONS_00095452/TCONS_00096376	ENSSSCT00000005271/ENSSSCT00000010229/ENSSSCT00000013322/ENSSSCT00000018768/ENSSSCT00000027462/ENSSSCT00000028216/ENSSSCT00000030802/ENSSSCT00000031202/ENSSSCT00000035973/TCONS_00006377/TCONS_00008348/TCONS_00008709/TCONS_00011158/TCONS_00016869/TCONS_00016870/TCONS_00020520/TCONS_00022468/TCONS_00031548/TCONS_00035410/TCONS_00035601/TCONS_00038058/TCONS_00041695/TCONS_00045630/TCONS_00049436/TCONS_00052677/TCONS_00052953/TCONS_00072603/TCONS_00073850/TCONS_00077645/TCONS_00081205/TCONS_00084552/TCONS_00085534/TCONS_00085544/TCONS_00090841/TCONS_00096230/TCONS_00100501/TCONS_00100528/TCONS_00100529
Biological Process	hormone secretion	0	1		TCONS_00048384
Biological Process	reproductive process	3	5	TCONS_00033609/TCONS_00057499/TCONS_00080151	TCONS_00003027/TCONS_00049436/TCONS_00053886/TCONS_00085534/TCONS_00085544
Biological	multicellular	18	37	TCONS_00004931/TCONS_00010673/TCONS_	ENSSSCT00000008247/ENSSSCT00000018501/ENSSSC

Process	organismal process			00012908/TCONS_00020459/TCONS_00028503/TCONS_00029075/TCONS_00032517/TCONS_00033609/TCONS_00042642/TCONS_00046541/TCONS_00049007/TCONS_00051311/TCONS_00057499/TCONS_00074295/TCONS_00090200/TCONS_00090930/TCONS_00095452/TCONS_00096376	T00000035973/TCONS_00003027/TCONS_00004822/TCONS_00006314/TCONS_00006316/TCONS_00007428/TCONS_00008709/TCONS_00011158/TCONS_00016869/TCONS_00016870/TCONS_00017991/TCONS_00024980/TCONS_00024981/TCONS_00028230/TCONS_00031548/TCONS_00035093/TCONS_00035601/TCONS_00038058/TCONS_00041695/TCONS_00045630/TCONS_00048384/TCONS_00049436/TCONS_00050770/TCONS_00052642/TCONS_00053886/TCONS_00058416/TCONS_00078029/TCONS_00081205/TCONS_00084498/TCONS_00085534/TCONS_00085544/TCONS_00092241/TCONS_00096230/TCONS_00100528/TCONS_00100529
Biological Process	biological regulation	45	77	ENSSSCT00000009859/ENSSSCT00000014241/ENSSSCT00000015384/ENSSSCT00000018381/ENSSSCT00000023699/ENSSSCT00000028237/ENSSSCT00000032662/ENSSSCT00000036440/TCONS_00000968/TCONS_00001840/TCONS_00003237/TCONS_00004931/TCONS_00009137/TCONS_00010673/TCONS_00012908/TCONS_00018210/TCONS_00018343/TCONS_00020459/TCONS_00021033/TCONS_00029075/TCONS_00032517/TCONS_00033609/TCONS_0003393	ENSSSCT00000002510/ENSSSCT00000007604/ENSSSCT00000008247/ENSSSCT00000009813/ENSSSCT00000011053/ENSSSCT00000015576/ENSSSCT00000017282/ENSSSCT00000025648/ENSSSCT00000026377/ENSSSCT00000033729/ENSSSCT00000034484/ENSSSCT00000034640/ENSSSCT00000035749/ENSSSCT00000035973/ENSSSCT00000035982/ENSSSCT00000036373/TCONS_00003027/TCONS_00003539/TCONS_00004822/TCONS_00007428/TCONS_00008348/TCONS_00008709/TCONS_00011158/TCONS_00015685/TCONS_00016536/TCONS_00017991/

				7/TCONS_00046541/TCONS_00046597/TCONS_00049007/TCONS_00049538/TCONS_00049672/TCONS_00051311/TCONS_00055773/TCONS_00057499/TCONS_00059949/TCONS_00063147/TCONS_00067733/TCONS_00074295/TCONS_00076786/TCONS_00080151/TCONS_00084266/TCONS_00087175/TCONS_00091479/TCONS_00094383/TCONS_00095452/TCONS_00096376/TCONS_00097159/TCONS_00099248	TCONS_00020520/TCONS_00021034/TCONS_00022468/TCONS_00024980/TCONS_00024981/TCONS_00025388/TCONS_00028230/TCONS_00028709/TCONS_00029348/TCONS_00031135/TCONS_00031328/TCONS_00031548/TCONS_00032179/TCONS_00035093/TCONS_00035410/TCONS_00038058/TCONS_00041695/TCONS_00042856/TCONS_00045347/TCONS_00045349/TCONS_00045630/TCONS_00048384/TCONS_00049436/TCONS_00049673/TCONS_00052642/TCONS_00052677/TCONS_00052953/TCONS_00054717/TCONS_00058416/TCONS_00065835/TCONS_00068370/TCONS_00072603/TCONS_00073850/TCONS_00076744/TCONS_00077645/TCONS_00081205/TCONS_00083743/TCONS_00084498/TCONS_00084552/TCONS_00085534/TCONS_00085544/TCONS_00086748/TCONS_00090841/TCONS_00092241/TCONS_00094370/TCONS_00096230/TCONS_00100501/TCONS_00100513/TCONS_00100515/TCONS_00100528/TCONS_00100529
Biological Process	growth	2	3	TCONS_00033609/TCONS_00096376	TCONS_00050770/TCONS_00100528/TCONS_00100529
Biological Process	cellular process	79	118	ENSSSCT00000000934/ENSSSCT00000002808/ENSSSCT00000006699/ENSSSCT00000007534/ENSSSCT00000009859/ENSSSCT00000011308/	ENSSSCT00000002510/ENSSSCT00000005271/ENSSSCT00000007604/ENSSSCT00000008247/ENSSSCT00000008947/ENSSSCT00000009813/ENSSSCT00000010229/ENS

ENSSSCT00000011527/	SSCT00000011053/ENSSSC
ENSSSCT00000013502/	T00000013322/ENSSSCT000
ENSSSCT00000013571/	00015061/ENSSSCT0000001
ENSSSCT00000014241/	5576/ENSSSCT00000017282/
ENSSSCT00000014538/	ENSSSCT00000017523/ENS
ENSSSCT00000015384/	SSCT00000018501/ENSSSC
ENSSSCT00000015449/	T00000018768/ENSSSCT000
ENSSSCT00000017392/	00019062/ENSSSCT0000002
ENSSSCT00000018381/	4741/ENSSSCT00000024980/
ENSSSCT00000019098/	ENSSSCT00000025648/ENS
ENSSSCT00000019681/	SSCT00000026377/ENSSSC
ENSSSCT00000026451/	T00000027462/ENSSSCT000
ENSSSCT00000027771/	00027869/ENSSSCT0000002
ENSSSCT00000029625/	8216/ENSSSCT00000028884/
ENSSSCT00000029905/	ENSSSCT00000031202/ENS
ENSSSCT00000031037/	SSCT00000032277/ENSSSC
ENSSSCT00000031122/	T00000033729/ENSSSCT000
ENSSSCT00000031428/	00034484/ENSSSCT0000003
ENSSSCT00000032662/	4640/ENSSSCT00000035032/
ENSSSCT00000034919/	ENSSSCT00000035749/ENS
ENSSSCT00000036434/	SSCT00000035973/ENSSSC
ENSSSCT00000036440/	T00000035982/ENSSSCT000
TCONS_00000968/TCO	00036290/ENSSSCT0000003
NS_00001139/TCONS_	6373/TCONS_00003027/TCO
00001840/TCONS_0000	NS_00004594/TCONS_00004
3237/TCONS_00004794	796/TCONS_00004822/TCO
/TCONS_00004931/TC	NS_00005241/TCONS_00006
ONS_00009137/TCONS	314/TCONS_00006316/TCO
_00012908/TCONS_000	NS_00006377/TCONS_00008
13396/TCONS_0001663	348/TCONS_00008709/TCO
7/TCONS_00016859/TC	NS_00011158/TCONS_00012
ONS_00018210/TCONS	424/TCONS_00013318/TCO
_00020459/TCONS_000	NS_00013451/TCONS_00013
24355/TCONS_0002521	452/TCONS_00014951/TCO
6/TCONS_00028863/TC	NS_00015685/TCONS_00016
ONS_00032517/TCONS	536/TCONS_00016869/TCO
_00033609/TCONS_000	NS_00016870/TCONS_00017
33937/TCONS_0003684	991/TCONS_00020222/TCO
2/TCONS_00042642/TC	NS_00020520/TCONS_00022
ONS_00045665/TCONS	007/TCONS_00022468/TCO
_00046597/TCONS_000	NS_00024980/TCONS_00024
49007/TCONS_0004953	981/TCONS_00025388/TCO
8/TCONS_00050395/TC	NS_00028230/TCONS_00029
ONS_00051311/TCONS	348/TCONS_00031328/TCO

				_00055773/TCONS_00057499/TCONS_00059949/TCONS_00062759/TCONS_00063147/TCONS_00064907/TCONS_00065482/TCONS_00067733/TCONS_00074295/TCONS_00075802/TCONS_00076786/TCONS_00080151/TCONS_00081239/TCONS_00083694/TCONS_00087175/TCONS_00087195/TCONS_00088728/TCONS_00090200/TCONS_00090930/TCONS_00094383/TCONS_00095452/TCONS_00096376/TCONS_00098418/TCONS_00099248	NS_00031548/TCONS_00032031/TCONS_00035093/TCONS_00035410/TCONS_00035601/TCONS_00036864/TCONS_00038058/TCONS_00041695/TCONS_00042856/TCONS_00045347/TCONS_00045349/TCONS_00045630/TCONS_00045691/TCONS_00045692/TCONS_00048384/TCONS_00049436/TCONS_00049673/TCONS_00050770/TCONS_00052642/TCONS_00052677/TCONS_00052953/TCONS_00053886/TCONS_00054717/TCONS_00058416/TCONS_00060620/TCONS_00068370/TCONS_00072603/TCONS_00073850/TCONS_00076744/TCONS_00077645/TCONS_00078029/TCONS_00081205/TCONS_00083743/TCONS_00084390/TCONS_00084498/TCONS_00084552/TCONS_00085534/TCONS_00085544/TCONS_00086748/TCONS_00090841/TCONS_00092241/TCONS_00094370/TCONS_00096230/TCONS_00098419/TCONS_00100494/TCONS_00100501/TCONS_00100510/TCONS_00100513/TCONS_00100515/TCONS_00100528/TCONS_00100529/TCONS_00100533
Biological Process	behavior	3	2	TCONS_00029075/TCONS_00090930/TCONS_00097159	TCONS_00038058/TCONS_00041695
Biological Process	single-organism process	65	94	ENSSSCT00000000934/ENSSSCT00000001927/ENSSSCT00000009163/ENSSSCT00000009164/ENSSSCT00000009859/	ENSSSCT00000002510/ENSSSCT00000006733/ENSSSCT00000007604/ENSSSCT00000008247/ENSSSCT00000009813/ENSSSCT00000010229/

ENSSSCT00000011308/ENSSSCT00000011527/ENSSSCT00000014241/ENSSSCT00000015384/ENSSSCT00000015449/ENSSSCT00000017392/ENSSSCT00000018381/ENSSSCT00000019098/ENSSSCT00000019681/ENSSSCT00000027771/ENSSSCT00000029625/ENSSSCT00000031037/ENSSSCT00000031428/ENSSSCT00000032662/ENSSSCT00000036434/ENSSSCT00000036440/TCONS_00001840/TCONS_00003237/TCONS_00004931/TCONS_00009137/TCONS_00010673/TCONS_00012908/TCONS_00014448/TCONS_00016637/TCONS_00018210/TCONS_00020459/TCONS_00025216/TCONS_00028503/TCONS_00028863/TCONS_00029075/TCONS_00032517/TCONS_00033609/TCONS_00033937/TCONS_00036842/TCONS_00042642/TCONS_00046541/TCONS_00049007/TCONS_00050395/TCONS_00051311/TCONS_00051555/TCONS_00052894/TCONS_00055773/TCONS_00057499/TCONS_00059949/TCONS_00062759/TCONS_00063147/TCONS_00067733/TCONS_00072374/TCONS_00074295/TCONS_00080151/TCONS_0008123	ENSSSCT00000011053/ENSSSCT00000015576/ENSSSCT00000017282/ENSSSCT00000018501/ENSSSCT00000025648/ENSSSCT00000026377/ENSSSCT00000027462/ENSSSCT00000027869/ENSSSCT00000028884/ENSSSCT00000033729/ENSSSCT00000034484/ENSSSCT00000034640/ENSSSCT00000035292/ENSSSCT00000035749/ENSSSCT00000035973/ENSSSCT00000035982/ENSSSCT00000036373/TCONS_00003027/TCONS_00003539/TCONS_00004594/TCONS_00004822/TCONS_00006314/TCONS_00006316/TCONS_00006377/TCONS_00007428/TCONS_00008348/TCONS_00008709/TCONS_00011158/TCONS_00012424/TCONS_00014951/TCONS_00016869/TCONS_00016870/TCONS_00017991/TCONS_00020222/TCONS_00020520/TCONS_00022468/TCONS_00024980/TCONS_00024981/TCONS_00025388/TCONS_00028230/TCONS_00031135/TCONS_00031328/TCONS_00031548/TCONS_00035093/TCONS_00035410/TCONS_00035601/TCONS_00036864/TCONS_00038058/TCONS_00041695/TCONS_00042856/TCONS_00045347/TCONS_00045349/TCONS_00045630/TCONS_00045691/TCONS_00045692/TCONS_00048384/TCONS_00049436/TCONS_00049673/TCONS_00050770/TCONS_00052642/TCONS_00052677/TCONS_00052

				9/TCONS_00083694/TCONS_00087195/TCONS_00090200/TCONS_00090930/TCONS_00091479/TCONS_00094383/TCONS_00095452/TCONS_00096376/TCONS_00097159	953/TCONS_00053886/TCONS_00054717/TCONS_00058416/TCONS_00060620/TCONS_00065835/TCONS_00066789/TCONS_00068370/TCONS_00072603/TCONS_00073850/TCONS_00076154/TCONS_00077645/TCONS_00078029/TCONS_00081205/TCONS_00083743/TCONS_00084498/TCONS_00085534/TCONS_00085544/TCONS_00086748/TCONS_00090841/TCONS_00092241/TCONS_00094370/TCONS_00096230/TCONS_00100501/TCONS_00100510/TCONS_00100528/TCONS_00100529
Biological Process	immune system process	9	10	ENSSSCT00000001329/ENSSSCT00000009461/ENSSSCT00000009859/ENSSSCT00000010832/ENSSSCT00000014800/TCONS_00001840/TCONS_00033937/TCONS_00049007/TCONS_00063147	ENSSSCT00000009813/ENSSSCT00000033729/ENSSSCT00000034640/ENSSSCT00000035973/TCONS_00004822/TCONS_00007428/TCONS_00031548/TCONS_00045630/TCONS_00085534/TCONS_00085544
Biological Process	locomotion	2	11	ENSSSCT00000029625/TCONS_00051311	ENSSSCT00000009813/ENSSSCT00000034640/TCONS_00014951/TCONS_00031548/TCONS_00038058/TCONS_00052953/TCONS_00054717/TCONS_00085534/TCONS_00085544/TCONS_00100528/TCONS_00100529
Biological Process	localization	27	33	ENSSSCT00000003939/ENSSSCT00000008741/ENSSSCT00000011308/ENSSSCT00000015449/ENSSSCT00000017392/ENSSSCT00000018381/ENSSSCT00000019098/ENSSSCT00000027232/ENSSSCT00000028237/	ENSSSCT00000009813/ENSSSCT00000015576/ENSSSCT00000027462/ENSSSCT00000027869/ENSSSCT00000030802/ENSSSCT00000034640/ENSSSCT00000035973/TCONS_00003539/TCONS_00004822/TCONS_00008348/TCONS_00008709/TCONS_00011

				ENSSSCT00000029625/ENSSSCT00000031428/ENSSSCT00000036440/ENSSSCT00000036536/TCONS_00007403/TCONS_00013396/TCONS_00018210/TCONS_00025216/TCONS_00028503/TCONS_00037492/TCONS_00046541/TCONS_00049538/TCONS_00050395/TCONS_00057499/TCONS_00063147/TCONS_00072374/TCONS_00080151/TCONS_00091479	158/TCONS_00014951/TCONS_00015685/TCONS_00020222/TCONS_00020520/TCONS_00022468/TCONS_00036864/TCONS_00038058/TCONS_00045630/TCONS_00048384/TCONS_00050770/TCONS_00052953/TCONS_00054717/TCONS_00058416/TCONS_00066789/TCONS_00068370/TCONS_00083743/TCONS_00084552/TCONS_00085544/TCONS_00096230/TCONS_00100528/TCONS_00100529
Biological Process	biological adhesion	4	9	ENSSSCT00000016780/ENSSSCT00000032593/TCONS_00074295/TCONS_00094383	ENSSSCT00000004436/ENSSSCT00000005288/ENSSSCT00000007604/ENSSSCT00000017016/ENSSSCT00000030852/TCONS_00008709/TCONS_00031548/TCONS_00035093/TCONS_00085544
Biological Process	reproduction	1	4	TCONS_00033609	ENSSSCT00000035973/TCONS_00053886/TCONS_00085534/TCONS_00085544
Biological Process	multi-organism process	2	5	TCONS_00003237/TCONS_00033609	TCONS_00049436/TCONS_00053886/TCONS_00073850/TCONS_00085544/TCONS_00100501
Biological Process	response to stimulus	25	54	ENSSSCT00000009461/ENSSSCT00000009859/ENSSSCT00000010832/ENSSSCT00000014241/ENSSSCT00000014800/ENSSSCT00000015384/ENSSSCT00000031972/ENSSSCT00000032662/TCONS_00001840/TCONS_00003237/TCONS_00009137/TCONS_00020459/TCONS_00029075/TCONS_00033609/TC	ENSSSCT00000002510/ENSSSCT00000005288/ENSSSCT00000007604/ENSSSCT00000008247/ENSSSCT00000009813/ENSSSCT00000011053/ENSSSCT00000017282/ENSSSCT00000025648/ENSSSCT00000026377/ENSSSCT00000033729/ENSSSCT00000034484/ENSSSCT00000034640/ENSSSCT00000035749/ENSSSCT00000035973/ENSSSCT00000035982/ENSSSCT000

				ONS_00033937/TCONS_00049007/TCONS_00051311/TCONS_00051555/TCONS_00057499/TCONS_00059949/TCONS_00076786/TCONS_00080151/TCONS_00084266/TCONS_00090200/TCONS_00096376	00036373/TCONS_00003027/TCONS_00004822/TCONS_00008348/TCONS_00011158/TCONS_00015685/TCONS_00016536/TCONS_00016869/TCONS_00020520/TCONS_00022468/TCONS_00024980/TCONS_00024981/TCONS_00025388/TCONS_00028230/TCONS_00031328/TCONS_00031548/TCONS_00035410/TCONS_00038058/TCONS_00041695/TCONS_00042856/TCONS_00045347/TCONS_00045349/TCONS_00045630/TCONS_00048384/TCONS_00049436/TCONS_00049673/TCONS_00052642/TCONS_00052953/TCONS_00054717/TCONS_00072603/TCONS_00084498/TCONS_00085534/TCONS_00085544/TCONS_00090841/TCONS_00092241/TCONS_00094370/TCONS_00100501/TCONS_00100528/TCONS_00100529
Biological Process	signaling	13	43	ENSSSCT00000009859/ENSSSCT00000014241/ENSSSCT00000015384/ENSSSCT00000032662/TCONS_00001840/TCONS_00003237/TCONS_00020459/TCONS_00033609/TCONS_00033937/TCONS_00049007/TCONS_00057499/TCONS_00074295/TCONS_00096376	ENSSSCT00000002510/ENSSSCT00000007604/ENSSSCT00000008247/ENSSSCT00000009813/ENSSSCT00000011053/ENSSSCT00000017282/ENSSSCT00000025648/ENSSSCT00000026377/ENSSSCT00000033729/ENSSSCT00000034484/ENSSSCT00000034640/ENSSSCT00000035749/ENSSSCT00000035973/ENSSSCT00000035982/ENSSSCT00000036373/TCONS_00011158/TCONS_00024980/TCONS_00024981/TCONS_00025388/TCONS_00028230/TCONS_00031328/TCONS_00031548/TCONS_00035410/TCO

					NS_00038058/TCONS_00041695/TCONS_00042856/TCONS_00045347/TCONS_00045349/TCONS_00048384/TCONS_00049436/TCONS_00049673/TCONS_00052642/TCONS_00054717/TCONS_00072603/TCONS_00084498/TCONS_00085534/TCONS_00085544/TCONS_00090841/TCONS_00092241/TCONS_00096230/TCONS_00100501/TCONS_00100528/TCONS_00100529
Molecular Function	catalytic activity	68	60	ENSSSCT00000000934/ENSSSCT00000001927/ENSSSCT00000002252/ENSSSCT00000006699/ENSSSCT00000006967/ENSSSCT00000009163/ENSSSCT00000009164/ENSSSCT00000009375/ENSSSCT00000010832/ENSSSCT00000011308/ENSSSCT00000013502/ENSSSCT00000013571/ENSSSCT00000014538/ENSSSCT00000015432/ENSSSCT00000015449/ENSSSCT00000018381/ENSSSCT00000019681/ENSSSCT00000019687/ENSSSCT00000022502/ENSSSCT00000023318/ENSSSCT00000023699/ENSSSCT00000027771/ENSSSCT00000028237/ENSSSCT00000029625/ENSSSCT00000029905/ENSSSCT00000030903/ENSSSCT00000031122/ENSSSCT00000033745/ENSSSCT00000036434/ENSSSCT00000036440/	ENSSSCT00000006733/ENSSSCT00000008247/ENSSSCT00000011053/ENSSSCT00000013322/ENSSSCT00000016707/ENSSSCT00000017523/ENSSSCT00000018236/ENSSSCT00000018501/ENSSSCT00000023398/ENSSSCT00000024741/ENSSSCT00000027462/ENSSSCT00000028542/ENSSSCT00000028884/ENSSSCT00000030802/ENSSSCT00000031950/ENSSSCT00000034484/ENSSSCT00000035032/ENSSSCT00000035292/ENSSSCT00000035982/TCONS_00002883/TCONS_00003027/TCONS_00004796/TCONS_00005241/TCONS_00006314/TCONS_00006316/TCONS_00008348/TCONS_00008709/TCONS_00011474/TCONS_00012424/TCONS_00013318/TCONS_00013452/TCONS_00014076/TCONS_00016536/TCONS_00022007/TCONS_00028230/TCONS_00029348/TCONS_00032031/TCONS_00041695/TCONS_00042856/TCONS_00048384/TCO

				TCONS_00001139/TCONS_00001244/TCONS_00004794/TCONS_00004931/TCONS_00007403/TCONS_00010927/TCONS_00012870/TCONS_00014448/TCONS_00016859/TCONS_00018210/TCONS_00020459/TCONS_00024355/TCONS_00028863/TCONS_00032517/TCONS_00033167/TCONS_00033609/TCONS_00036842/TCONS_00037171/TCONS_00042642/TCONS_00045665/TCONS_00049007/TCONS_00051555/TCONS_00052894/TCONS_00055773/TCONS_00059278/TCONS_00059949/TCONS_00064907/TCONS_00065482/TCONS_00067733/TCONS_00075802/TCONS_00081239/TCONS_00083694/TCONS_00088728/TCONS_00090200/TCONS_00090930/TCONS_00096376/TCONS_00098418/TCONS_00099248	NS_00049436/TCONS_00052677/TCONS_00052953/TCONS_00068370/TCONS_00076154/TCONS_00078029/TCONS_00083246/TCONS_00084390/TCONS_00084498/TCONS_00085534/TCONS_00085544/TCONS_00086748/TCONS_00094370/TCONS_00098419/TCONS_00100494/TCONS_00100501/TCONS_00100510/TCONS_00100528/TCONS_00100529/TCONS_00100533
Molecular Function	binding	84	113	ENSSSCT00000000934/ENSSSCT00000002812/ENSSSCT00000003939/ENSSSCT00000004174/ENSSSCT00000006226/ENSSSCT00000006699/ENSSSCT00000006706/ENSSSCT00000007206/ENSSSCT00000007534/ENSSSCT00000008741/ENSSSCT00000010013/ENSSSCT00000010832/ENSSSCT00000011527/	ENSSSCT00000000144/ENSSSCT00000002510/ENSSSCT00000002650/ENSSSCT00000002713/ENSSSCT00000004436/ENSSSCT00000005271/ENSSSCT00000005288/ENSSSCT00000005633/ENSSSCT00000006644/ENSSSCT00000006733/ENSSSCT00000007604/ENSSSCT00000008247/ENSSSCT00000008947/ENSSSCT00000010229/ENSSSCT00000011679/ENSSSCT000

ENSSSCT00000012400/ENSSSCT00000012465/ENSSSCT00000013502/ENSSSCT00000014241/ENSSSCT00000014538/ENSSSCT00000015432/ENSSSCT00000023318/ENSSSCT00000025191/ENSSSCT00000027232/ENSSSCT00000030903/ENSSSCT00000031037/ENSSSCT00000031122/ENSSSCT00000034919/ENSSSCT00000036440/ENSSSCT00000036536/TCONS_00000968/TCONS_00001840/TCONS_00004931/TCONS_00010673/TCONS_00012870/TCONS_00012908/TCONS_00013396/TCONS_00014448/TCONS_00016637/TCONS_00018210/TCONS_00018343/TCONS_00020459/TCONS_00021033/TCONS_00024355/TCONS_00025216/TCONS_00028187/TCONS_00028863/TCONS_00029075/TCONS_00032517/TCONS_00033167/TCONS_00033609/TCONS_00033937/TCONS_00034199/TCONS_00037171/TCONS_00045665/TCONS_00046597/TCONS_00049007/TCONS_00049538/TCONS_00049672/TCONS_00051311/TCONS_00051555/TCONS_00052894/TCONS_00055773/TCONS_00057499/TCONS_00058345/TCONS_00059949/TC	00013322/ENSSSCT00000013323/ENSSSCT00000015061/ENSSSCT00000015660/ENSSSCT00000015663/ENSSSCT00000017282/ENSSSCT00000017523/ENSSSCT00000018236/ENSSSCT00000018768/ENSSSCT00000019062/ENSSSCT00000019601/ENSSSCT00000024980/ENSSSCT00000027235/ENSSSCT00000028216/ENSSSCT00000028542/ENSSSCT00000028884/ENSSSCT00000030802/ENSSSCT00000031202/ENSSSCT00000031950/ENSSSCT00000032277/ENSSSCT00000032649/ENSSSCT00000034484/ENSSSCT00000035485/ENSSSCT00000035876/ENSSSCT00000035973/ENSSSCT00000036290/TCONS_00003027/TCONS_00004822/TCONS_00007428/TCONS_00008348/TCONS_00008709/TCONS_00011158/TCONS_00012424/TCONS_00014951/TCONS_00015685/TCONS_00016536/TCONS_00016869/TCONS_00017991/TCONS_00020520/TCONS_00021034/TCONS_00022007/TCONS_00024980/TCONS_00024981/TCONS_00025388/TCONS_00028230/TCONS_00029348/TCONS_00029651/TCONS_00031135/TCONS_00031328/TCONS_00031548/TCONS_00032179/TCONS_00035093/TCONS_00035410/TCONS_00035601/TCONS_00037624/TCONS_00038058/TCONS_00041695/TCONS_00042856/TCONS_00045349/TCONS_00045630/TCO

				ONS_00062759/TCONS_00063147/TCONS_00064152/TCONS_00064907/TCONS_00067733/TCONS_00074295/TCONS_00075802/TCONS_00080151/TCONS_00081239/TCONS_00083694/TCONS_00084266/TCONS_00087175/TCONS_00087195/TCONS_00090200/TCONS_00090930/TCONS_00095452/TCONS_00096376/TCONS_00097159/TCONS_00097868/TCONS_00099248	NS_00045691/TCONS_00045692/TCONS_00048384/TCONS_00049436/TCONS_00049673/TCONS_00052642/TCONS_00052677/TCONS_00052953/TCONS_00054717/TCONS_00058416/TCONS_00059260/TCONS_00060021/TCONS_00060620/TCONS_00066789/TCONS_00072603/TCONS_00073850/TCONS_00076154/TCONS_00076744/TCONS_00077645/TCONS_00078029/TCONS_00081205/TCONS_00083246/TCONS_00084498/TCONS_00084552/TCONS_00085534/TCONS_00085544/TCONS_00086748/TCONS_00089453/TCONS_00090841/TCONS_00092241/TCONS_00094370/TCONS_00096230/TCONS_00100501/TCONS_00100510/TCONS_00100513/TCONS_00100515/TCONS_00100528/TCONS_00100529
Molecular Function	nucleic acid binding transcription factor activity	4	18	ENSSSCT00000014241/ENSSSCT00000031037/TCONS_00049672/TCONS_00095452	ENSSSCT00000002510/ENSSSCT00000002650/ENSSSCT00000013323/ENSSSCT00000015660/ENSSSCT00000015663/ENSSSCT00000017282/ENSSSCT00000035485/ENSSSCT00000036290/TCONS_00007428/TCONS_00017991/TCONS_00024980/TCONS_00024981/TCONS_00038058/TCONS_00049673/TCONS_00052642/TCONS_00058416/TCONS_00076744/TCONS_00092241
Molecular Function	electron carrier activity	1	0	TCONS_00072374	

Molecular Function	channel regulator activity	1	1	TCONS_00033609	TCONS_00008348
Molecular Function	protein binding transcription factor activity	2	5	TCONS_00018343/TCONS_00095452	ENSSSCT00000036290/TCONS_00020520/TCONS_00048384/TCONS_00052953/TCONS_00092241
Molecular Function	transporter activity	9	10	ENSSSCT00000015449/ENSSSCT00000017392/ENSSSCT00000019098/ENSSSCT00000031428/ENSSSCT00000036440/TCONS_00046541/TCONS_00057499/TCONS_00080151/TCONS_00091479	ENSSSCT00000015576/ENSSSCT00000027869/TCONS_00004822/TCONS_00020222/TCONS_00022468/TCONS_00036864/TCONS_00045630/TCONS_00050770/TCONS_00068370/TCONS_00083743
Molecular Function	structural molecule activity	1	7	ENSSSCT00000002808	ENSSSCT00000008947/ENSSSCT00000018768/ENSSSCT00000019062/ENSSSCT00000027298/ENSSSCT00000028216/ENSSSCT00000031509/TCONS_00016869
Molecular Function	guanyl-nucleotide exchange factor activity	1	1	TCONS_00009137	TCONS_00073850
Molecular Function	molecular function regulator	7	4	ENSSSCT00000014800/ENSSSCT00000026451/TCONS_00009137/TCONS_00033609/TCONS_00080151/TCONS_00084266/TCONS_00094383	ENSSSCT00000025648/TCONS_00008348/TCONS_00032179/TCONS_00073850
Molecular Function	enzyme regulator activity	5	2	ENSSSCT00000014800/ENSSSCT00000026451/TCONS_00080151/TCONS_00084266/TCONS_00094383	ENSSSCT00000025648/TCONS_00032179

Molecular Function	molecular transducer activity	5	18	ENSSSCT00000009859/ENSSSCT00000014241/ENSSSCT00000032662/TCONS_00003237/TCONS_00028503	ENSSSCT00000002510/ENSSSCT00000008247/ENSSSCT00000017282/ENSSSCT00000033729/ENSSSCT00000034484/ENSSSCT00000035982/TCONS_00011158/TCONS_00020520/TCONS_00031548/TCONS_00038058/TCONS_00041695/TCONS_00042856/TCONS_00048384/TCONS_00049436/TCONS_00049673/TCONS_00083743/TCONS_00085534/TCONS_00085544
Cellular Component	organelle	79	81	ENSSSCT00000002252/ENSSSCT00000002808/ENSSSCT00000007534/ENSSSCT00000009461/ENSSSCT00000011308/ENSSSCT00000013502/ENSSSCT00000014241/ENSSSCT00000015449/ENSSSCT00000016961/ENSSSCT00000018381/ENSSSCT00000019681/ENSSSCT00000019687/ENSSSCT00000027771/ENSSSCT00000028237/ENSSSCT00000029905/ENSSSCT00000031037/ENSSSCT00000031122/ENSSSCT00000033745/ENSSSCT00000035069/ENSSSCT00000036352/ENSSSCT00000036434/ENSSSCT00000036440/TCONS_00000968/TCONS_00001244/TCONS_00001840/TCONS_00004794/TCONS_00004931/TCONS_00007403/TCONS_00009137/TCONS_00010673/TCONS_00010927/TCONS_00013396/TCONS_00014448/TC	ENSSSCT00000002510/ENSSSCT00000002713/ENSSSCT00000005271/ENSSSCT00000008947/ENSSSCT00000013323/ENSSSCT00000015660/ENSSSCT00000015663/ENSSSCT00000017282/ENSSSCT00000018768/ENSSSCT00000019062/ENSSSCT00000019601/ENSSSCT00000027298/ENSSSCT00000027462/ENSSSCT00000028216/ENSSSCT00000028542/ENSSSCT00000028884/ENSSSCT00000029040/ENSSSCT00000030802/ENSSSCT00000031509/ENSSSCT00000035485/ENSSSCT00000035973/ENSSSCT00000036290/TCONS_00003027/TCONS_00003539/TCONS_00004594/TCONS_00004796/TCONS_00006377/TCONS_00007428/TCONS_00008348/TCONS_00008709/TCONS_00011115/TCONS_00011158/TCONS_00012424/TCONS_00013318/TCONS_00014951/TCONS_00015685/TCONS_00016536/TCONS_00017991/TCONS_00020520/TCONS_00022007/TCONS_00022468/

				ONS_00016637/TCONS_00018210/TCONS_00018343/TCONS_00020459/TCONS_00025216/TCONS_00028187/TCONS_00028503/TCONS_00028863/TCONS_00029075/TCONS_00033167/TCONS_00033609/TCONS_00034199/TCONS_00036842/TCONS_00037171/TCONS_00042642/TCONS_00046597/TCONS_00049007/TCONS_00049538/TCONS_00049672/TCONS_00050395/TCONS_00051311/TCONS_00051555/TCONS_00055773/TCONS_00057499/TCONS_00059278/TCONS_00059949/TCONS_00063147/TCONS_00064907/TCONS_00067733/TCONS_00072374/TCONS_00074295/TCONS_00076786/TCONS_00080151/TCONS_00081239/TCONS_00084266/TCONS_00087175/TCONS_00087195/TCONS_00088728/TCONS_00090200/TCONS_00091479/TCONS_00094383/TCONS_00095452/TCONS_00096376/TCONS_00097159/TCONS_00097868/TCONS_00099248	TCONS_00024980/TCONS_00024981/TCONS_00025388/TCONS_00028230/TCONS_00031135/TCONS_00031328/TCONS_00032179/TCONS_00035601/TCONS_00038058/TCONS_00041695/TCONS_00042856/TCONS_00045630/TCONS_00048384/TCONS_00049436/TCONS_00049673/TCONS_00050770/TCONS_00052642/TCONS_00052677/TCONS_00052953/TCONS_00058416/TCONS_00060620/TCONS_00068370/TCONS_00072603/TCONS_00073850/TCONS_00076154/TCONS_00076744/TCONS_00077645/TCONS_00081205/TCONS_00084390/TCONS_00084552/TCONS_00085505/TCONS_00085544/TCONS_00086748/TCONS_00090841/TCONS_00092241/TCONS_00096230/TCONS_00100501/TCONS_00100510/TCONS_00100513/TCONS_00100515
Cellular Component	cell part	91	106	ENSSSCT00000001329/ENSSSCT00000002252/ENSSSCT00000002808/ENSSSCT00000007534/ENSSSCT00000008741/ENSSSCT00000009461/ENSSSCT00000011308/	ENSSSCT00000002510/ENSSSCT00000002713/ENSSSCT00000005271/ENSSSCT00000008247/ENSSSCT00000008947/ENSSSCT00000011053/ENSSSCT00000013322/ENSSSCT00000013323/ENSSSC

ENSSSCT00000011527/ENSSSCT00000013502/ENSSSCT00000013571/ENSSSCT00000014241/ENSSSCT00000015449/ENSSSCT00000016961/ENSSSCT00000017392/ENSSSCT00000018381/ENSSSCT00000019098/ENSSSCT00000019681/ENSSSCT00000019687/ENSSSCT00000026451/ENSSSCT00000027771/ENSSSCT00000028237/ENSSSCT00000029625/ENSSSCT00000029905/ENSSSCT00000031037/ENSSSCT00000031122/ENSSSCT00000031428/ENSSSCT00000033745/ENSSSCT00000035069/ENSSSCT00000036352/ENSSSCT00000036434/ENSSSCT00000036440/ENSSSCT00000036536/TCONS_00000968/TCONS_00001244/TCONS_00001840/TCONS_00004794/TCONS_00004931/TCONS_00007403/TCONS_00009137/TCONS_00010673/TCONS_00010927/TCONS_00012870/TCONS_00012908/TCONS_00013396/TCONS_00014448/TCONS_00016637/TCONS_00018210/TCONS_00018343/TCONS_00020459/TCONS_00025216/TCONS_00028187/TCONS_00028503/TCONS_00028863/TCONS_00029075/TCONS_00033167/TCONS_000	T00000015576/ENSSSCT00000015660/ENSSSCT00000015663/ENSSSCT00000017016/ENSSSCT00000017282/ENSSSCT00000018768/ENSSSCT00000019062/ENSSSCT00000019601/ENSSSCT00000026377/ENSSSCT00000027298/ENSSSCT00000027462/ENSSSCT00000027869/ENSSSCT00000028216/ENSSSCT00000028542/ENSSSCT00000028884/ENSSSCT00000029040/ENSSSCT00000030802/ENSSSCT00000031509/ENSSSCT00000032277/ENSSSCT00000035485/ENSSSCT00000035973/ENSSSCT00000036290/ENSSSCT00000036373/TCONS_00003027/TCONS_00003539/TCONS_00004594/TCONS_00004796/TCONS_00004822/TCONS_00006377/TCONS_00007428/TCONS_00008709/TCONS_00011115/TCONS_00011158/TCONS_00012424/TCONS_00013318/TCONS_00014951/TCONS_00015685/TCONS_00016536/TCONS_00017991/TCONS_00020520/TCONS_00022007/TCONS_00022468/TCONS_00024980/TCONS_00024981/TCONS_00025388/TCONS_00028230/TCONS_00028709/TCONS_00029348/TCONS_00031135/TCONS_00031328/TCONS_00032179/TCONS_00035093/TCONS_00035410/TCONS_00035601/TCONS_00038058/TCONS_00041695/TCONS_00042603/TCONS_00042856/TCONS_00045347/TCONS_00045349/TCONS_00045

				33609/TCONS_00033937/TCONS_00036842/TCONS_00042642/TCONS_00046541/TCONS_00046597/TCONS_00049007/TCONS_00049538/TCONS_00049672/TCONS_00050395/TCONS_00051311/TCONS_00051555/TCONS_00055773/TCONS_00057499/TCONS_00059278/TCONS_00059949/TCONS_00063147/TCONS_00064907/TCONS_00067733/TCONS_00072374/TCONS_00074295/TCONS_00076786/TCONS_00080151/TCONS_00080339/TCONS_00081239/TCONS_00083694/TCONS_00084266/TCONS_00087175/TCONS_00087195/TCONS_00090200/TCONS_00091479/TCONS_00094383/TCONS_00095452/TCONS_00096376/TCONS_00097868/TCONS_00099248	630/TCONS_00045691/TCONS_00045692/TCONS_00048384/TCONS_00049436/TCONS_00049673/TCONS_00050770/TCONS_00052642/TCONS_00052677/TCONS_00052953/TCONS_00054717/TCONS_00058416/TCONS_00060021/TCONS_00060620/TCONS_00065835/TCONS_00072603/TCONS_00073850/TCONS_00076154/TCONS_00076744/TCONS_00077645/TCONS_00078029/TCONS_00081205/TCONS_00084390/TCONS_00084498/TCONS_00084552/TCONS_00085505/TCONS_00085534/TCONS_00085544/TCONS_00086748/TCONS_00090841/TCONS_00092241/TCONS_00096230/TCONS_00100501/TCONS_00100510/TCONS_00100513/TCONS_00100515/TCONS_00100528/TCONS_00100529
Cellular Component	cell	91	106	ENSSSCT00000001329/ENSSSCT00000002252/ENSSSCT00000002808/ENSSSCT00000007534/ENSSSCT00000008741/ENSSSCT00000009461/ENSSSCT00000011308/ENSSSCT00000011527/ENSSSCT00000013502/ENSSSCT00000013571/ENSSSCT00000014241/ENSSSCT00000015449/ENSSSCT00000016961/ENSSSCT00000017392/ENSSSCT00000018381/	ENSSSCT00000002510/ENSSSCT00000002713/ENSSSCT00000005271/ENSSSCT00000008247/ENSSSCT00000008947/ENSSSCT00000011053/ENSSSCT00000013322/ENSSSCT00000013323/ENSSSCT00000015576/ENSSSCT00000015660/ENSSSCT00000015663/ENSSSCT00000017016/ENSSSCT00000017282/ENSSSCT00000018768/ENSSSCT00000019062/ENSSSCT00000019601/ENSSSCT00000026377/ENSSSCT00000027298/

ENSSSCT00000019098/	ENSSSCT00000027462/ENS
ENSSSCT00000019681/	SSCT00000027869/ENSSSC
ENSSSCT00000019687/	T00000028216/ENSSSCT000
ENSSSCT00000026451/	00028542/ENSSSCT0000002
ENSSSCT00000027771/	8884/ENSSSCT00000029040/
ENSSSCT00000028237/	ENSSSCT00000030802/ENS
ENSSSCT00000029625/	SSCT00000031509/ENSSSC
ENSSSCT00000029905/	T00000032277/ENSSSCT000
ENSSSCT00000031037/	00035485/ENSSSCT0000003
ENSSSCT00000031122/	5973/ENSSSCT00000036290/
ENSSSCT00000031428/	ENSSSCT00000036373/TCO
ENSSSCT00000033745/	NS_00003027/TCONS_00003
ENSSSCT00000035069/	539/TCONS_00004594/TCO
ENSSSCT00000036352/	NS_00004796/TCONS_00004
ENSSSCT00000036434/	822/TCONS_00006377/TCO
ENSSSCT00000036440/	NS_00007428/TCONS_00008
ENSSSCT00000036536/	709/TCONS_00011115/TCO
TCONS_00000968/TCO	NS_00011158/TCONS_00012
NS_00001244/TCONS_	424/TCONS_00013318/TCO
00001840/TCONS_0000	NS_00014951/TCONS_00015
4794/TCONS_00004931	685/TCONS_00016536/TCO
/TCONS_00007403/TC	NS_00017991/TCONS_00020
ONS_00009137/TCONS	520/TCONS_00022007/TCO
_00010673/TCONS_000	NS_00022468/TCONS_00024
10927/TCONS_0001287	980/TCONS_00024981/TCO
0/TCONS_00012908/TC	NS_00025388/TCONS_00028
ONS_00013396/TCONS	230/TCONS_00028709/TCO
_00014448/TCONS_000	NS_00029348/TCONS_00031
16637/TCONS_0001821	135/TCONS_00031328/TCO
0/TCONS_00018343/TC	NS_00032179/TCONS_00035
ONS_00020459/TCONS	093/TCONS_00035410/TCO
_00025216/TCONS_000	NS_00035601/TCONS_00038
28187/TCONS_0002850	058/TCONS_00041695/TCO
3/TCONS_00028863/TC	NS_00042603/TCONS_00042
ONS_00029075/TCONS	856/TCONS_00045347/TCO
_00033167/TCONS_000	NS_00045349/TCONS_00045
33609/TCONS_0003393	630/TCONS_00045691/TCO
7/TCONS_00036842/TC	NS_00045692/TCONS_00048
ONS_00042642/TCONS	384/TCONS_00049436/TCO
_00046541/TCONS_000	NS_00049673/TCONS_00050
46597/TCONS_0004900	770/TCONS_00052642/TCO
7/TCONS_00049538/TC	NS_00052677/TCONS_00052
ONS_00049672/TCONS	953/TCONS_00054717/TCO
_00050395/TCONS_000	NS_00058416/TCONS_00060

				51311/TCONS_00051555/TCONS_00055773/TCONS_00057499/TCONS_00059278/TCONS_00059949/TCONS_00063147/TCONS_00064907/TCONS_00067733/TCONS_00072374/TCONS_00074295/TCONS_00076786/TCONS_00080151/TCONS_00080339/TCONS_00081239/TCONS_00083694/TCONS_00084266/TCONS_00087175/TCONS_00087195/TCONS_00090200/TCONS_00091479/TCONS_00094383/TCONS_00095452/TCONS_00096376/TCONS_00097868/TCONS_00099248	021/TCONS_00060620/TCONS_00065835/TCONS_00072603/TCONS_00073850/TCONS_00076154/TCONS_00076744/TCONS_00077645/TCONS_00078029/TCONS_00081205/TCONS_00084390/TCONS_00084498/TCONS_00084552/TCONS_00085505/TCONS_00085534/TCONS_00085544/TCONS_00086748/TCONS_00090841/TCONS_00092241/TCONS_00096230/TCONS_00100501/TCONS_00100510/TCONS_00100513/TCONS_00100515/TCONS_00100528/TCONS_00100529
Cellular Component	organelle part	28	31	TCONS_00001840/TCONS_00004931/TCONS_00007403/TCONS_00014448/TCONS_00016637/TCONS_00018210/TCONS_00018343/TCONS_00020459/TCONS_00025216/TCONS_00028863/TCONS_00033609/TCONS_00042642/TCONS_00049672/TCONS_00050395/TCONS_00051311/TCONS_00051555/TCONS_00055773/TCONS_00057499/TCONS_00059278/TCONS_00059949/TCONS_00063147/TCONS_00064907/TCONS_00072374/TCONS_00087175/TCONS_00087195/TCONS_00090200/TCONS_00094383/TCONS_00096376	ENSSSCT00000005271/ENSSSCT00000013323/ENSSSCT00000036290/TCONS_00003027/TCONS_00004594/TCONS_00006377/TCONS_00007428/TCONS_00008709/TCONS_00012424/TCONS_00015685/TCONS_00016536/TCONS_00022468/TCONS_00031328/TCONS_00035601/TCONS_00038058/TCONS_00041695/TCONS_00045630/TCONS_00049673/TCONS_00052642/TCONS_00052953/TCONS_00058416/TCONS_00068370/TCONS_00073850/TCONS_00076154/TCONS_00077645/TCONS_00081205/TCONS_00084552/TCONS_00086748/TCONS_00092241/TCONS_00096230/TCONS_00100501

Cellular Component	nucleoid	2	0	TCONS_00020459/TCONS_00046597	
Cellular Component	membrane-enclosed lumen	12	13	TCONS_00001840/TCONS_00004931/TCONS_00007403/TCONS_00014448/TCONS_00018343/TCONS_00033609/TCONS_00049672/TCONS_00055773/TCONS_00063147/TCONS_00072374/TCONS_00087175/TCONS_00096376	ENSSSCT00000005271/ENSSSCT00000013323/ENSSSCT00000036290/TCONS_00003027/TCONS_00015685/TCONS_00016536/TCONS_00038058/TCONS_00049673/TCONS_00052642/TCONS_00052953/TCONS_00073850/TCONS_00092241/TCONS_00100501
Cellular Component	collagen trimer	0	2		TCONS_00016869/TCONS_00016870
Cellular Component	membrane part	13	24	TCONS_00003237/TCONS_00028503/TCONS_00036842/TCONS_00037492/TCONS_00046541/TCONS_00049672/TCONS_00051555/TCONS_00057499/TCONS_00063147/TCONS_00074295/TCONS_00080151/TCONS_00087195/TCONS_00091479	TCONS_00004822/TCONS_00008709/TCONS_00011158/TCONS_00014951/TCONS_00020222/TCONS_00022468/TCONS_00031548/TCONS_00035093/TCONS_00049436/TCONS_00049673/TCONS_00050770/TCONS_00053886/TCONS_00054717/TCONS_00058416/TCONS_00066789/TCONS_00068370/TCONS_00076154/TCONS_00077645/TCONS_00083743/TCONS_00084552/TCONS_00085534/TCONS_00085544/TCONS_00096230/TCONS_00098627
Cellular Component	extracellular matrix	1	7	TCONS_00094383	ENSSSCT00000007604/ENSSSCT00000031950/TCONS_00014951/TCONS_00016869/TCONS_00016870/TCONS_00028230/TCONS_00083246
Cellular Component	extracellular matrix component	1	3	TCONS_00094383	TCONS_00016869/TCONS_00016870/TCONS_00028230

Cellular Component	cell junction	3	6	TCONS_00057499/TCONS_00074295/TCONS_00097868	TCONS_00008709/TCONS_00045691/TCONS_00045692/TCONS_00085544/TCONS_00096230/TCONS_00098627
Cellular Component	membrane	31	42	ENSSSCT00000001329/ENSSSCT00000031428/TCONS_00000968/TCONS_00001840/TCONS_00003237/TCONS_00009137/TCONS_00014448/TCONS_00018210/TCONS_00020459/TCONS_00025216/TCONS_00028503/TCONS_00033609/TCONS_00033937/TCONS_00036842/TCONS_00037492/TCONS_00042642/TCONS_00046541/TCONS_00049007/TCONS_00049672/TCONS_00051555/TCONS_00055773/TCONS_00057499/TCONS_00059278/TCONS_00063147/TCONS_00072374/TCONS_00074295/TCONS_00080151/TCONS_00087195/TCONS_00090200/TCONS_00091479/TCONS_00097868	ENSSSCT00000002713/ENSSSCT00000008247/ENSSSCT00000011053/ENSSSCT00000015576/ENSSSCT00000017016/ENSSSCT00000027869/ENSSSCT00000036373/TCONS_00003539/TCONS_00004822/TCONS_00008348/TCONS_00008709/TCONS_00011158/TCONS_00012424/TCONS_00014951/TCONS_00020222/TCONS_00022468/TCONS_00025388/TCONS_00031328/TCONS_00031548/TCONS_00035093/TCONS_00045630/TCONS_00045691/TCONS_00045692/TCONS_00049436/TCONS_00049673/TCONS_00050770/TCONS_00052953/TCONS_00053886/TCONS_00054717/TCONS_00058416/TCONS_00066789/TCONS_00068370/TCONS_00073850/TCONS_00076154/TCONS_00077645/TCONS_00083743/TCONS_00084552/TCONS_00085534/TCONS_00085544/TCONS_00096230/TCONS_00098627/TCONS_00100501
Cellular Component	macromolecular complex	24	30	ENSSSCT00000001329/ENSSSCT00000002808/ENSSSCT00000008741/ENSSSCT00000013571/ENSSSCT00000017392/ENSSSCT00000019098/ENSSSCT00000026451/ENSSSCT00000035069/ENSSSCT00000036352/ENSSSCT00000036536/	ENSSSCT00000005271/ENSSSCT00000008247/ENSSSCT00000008947/ENSSSCT00000018768/ENSSSCT00000019062/ENSSSCT00000019601/ENSSSCT00000027298/ENSSSCT00000027462/ENSSSCT00000028216/ENSSSCT00000030802/ENSSSCT00000031509/ENSSSCT00000032277/

				TCONS_00000968/TCONS_00004931/TCONS_00012908/TCONS_00018210/TCONS_00018343/TCONS_00028187/TCONS_00055773/TCONS_00063147/TCONS_00072374/TCONS_00080151/TCONS_00083694/TCONS_00087175/TCONS_00091479/TCONS_00096376	ENSSSCT00000035973/ENSSSCT00000036290/TCONS_00006377/TCONS_00007428/TCONS_00016536/TCONS_00016869/TCONS_00016870/TCONS_00022007/TCONS_00049436/TCONS_00052642/TCONS_00065835/TCONS_00073850/TCONS_00081205/TCONS_00084498/TCONS_00086748/TCONS_00092241/TCONS_00096230/TCONS_00100501
Cellular Component	synapse part	0	2		TCONS_00042856/TCONS_00096230
Cellular Component	synapse	0	2		TCONS_00042856/TCONS_00096230
Cellular Component	extracellular region part	1	9	ENSSSCT00000014800	ENSSSCT00000005288/ENSSSCT00000007604/ENSSSCT00000009813/ENSSSCT00000031950/ENSSSCT00000034640/TCONS_00031548/TCONS_00045630/TCONS_00054717/TCONS_00085544
Cellular Component	extracellular region	2	10	ENSSSCT00000014800/ENSSSCT00000031972	ENSSSCT00000004436/ENSSSCT00000005288/ENSSSCT00000007604/ENSSSCT00000009813/ENSSSCT00000031950/ENSSSCT00000034640/TCONS_00031548/TCONS_00045630/TCONS_00054717/TCONS_00085544
